# Influence of mental health literacy on help-seeking behaviour for mental health problems in the Swiss young adult community: a cohort and longitudinal case–control study

**DOI:** 10.1007/s00406-022-01483-9

**Published:** 2022-09-11

**Authors:** N. Osman, C. Michel, B. G. Schimmelmann, L. Schilbach, E. Meisenzahl, F. Schultze-Lutter

**Affiliations:** 1 Department of Psychiatry and Psychotherapy/LVR-Klinikum Düsseldorf, Medical Faculty, Heinrich-Heine-University, Bergische Landstraße 2, 40629 Düsseldorf, Germany; 2 University Hospital of Child and Adolescent Psychiatry and Psychotherapy, University of Bern, Bern, Switzerland; 3 University Hospital of Child and Adolescent Psychiatry, University Hospital Hamburg-Eppendorf, Hamburg, Germany; 4 Medical Faculty, Ludwig Maximilians Universität, Munich, Germany; 5 Department of Psychology, Faculty of Psychology, Airlangga University, Surabaya, Indonesia

**Keywords:** Mental health literacy, Active help-seeking behaviour, Mental health problems/disorders, Longitudinal community study

## Abstract

Poor knowledge about mental health disorders and their treatment likely contributes to the large treatment gap reported for mental health problems. Therefore, we studied the association between mental health literacy (MHL) and active help-seeking in a community sample. Participants were recruited from an add-on questionnaire study to the ‘Bern Epidemiological At-Risk’ (BEAR) study on 16–40-year-old community subjects of the Swiss canton Bern. At baseline, data of *N* = 1504, and at 3-year follow-up, data of *N* = 535 were available. Based on an unlabelled case vignette (on depression or schizophrenia), MHL was assessed by the questionnaire of Angermeyer and colleagues. Cross-sectional and longitudinal baseline predictors of help-seeking were analysed using path analyses. Additionally, sensitivity analyses of the prospective model were computed for sex, vignette, and baseline mental health problems/disorders. Cross-sectionally, help-seeking was associated with non-endorsement of biogenetic causal explanations, presence of mental health problems/disorders, help-seeking before baseline, poorer functioning, and lower health satisfaction. The prospective model was similar; yet, help-seeking at follow-up was associated with endorsements of the causal explanation ‘biogenetics’ and, additionally, ‘childhood trauma’ but not the presence of baseline mental health problems/disorders. Sensitivity analyses revealed a significant impact on sex, vignette, and mental health problems/disorders. For example, actual functional problems were predictive in males, while health satisfaction was predictive in females. Our findings indicate that future studies on drivers of help-seeking should assess very large community samples with case vignettes on different mental disorders to examine appropriate subgroups and their likely interaction to address group-specific factors in awareness campaigns.

**Supplementary Information:**

The online version contains supplementary material available at 10.1007/s00406-022-01483-9.

## Introduction

 Approximately one in three people worldwide suffer from a mental disorder at some point in their lives [1] but only a minority seek professional help for it—often with considerable delay [2, 3]. This treatment delay has been linked to a worse outcome and, relatedly, higher burden and costs [4, 5]. To improve help-seeking for mental health problems (henceforth: help-seeking), its determinants need to be understood.

Poor mental health literacy (MHL) is considered one of the main reasons for help-seeking delays [6]. MHL was defined as “knowledge and beliefs about mental disorders which aid their recognition, management or prevention” [7], whereby ‘accurate’ beliefs that reflect the present state of knowledge indicate better MHL. Depending on the type of mental health (MH) problems/disorders, the correct identification of MH disorders and biogenetic causal explanations have been linked with higher help-seeking intentions for own MH problems/disorders [8–10], whereby biogenetic causal explanations were additionally associated with assumed poorer prognosis [11]. Furthermore, depression was reported to be correctly recognized more often than psychosis [12–16], and was attributed more frequently to psychosocial causes, whereas psychosis was attributed more frequently to biogenetic causes [12, 17, 18]. Both biogenetic and psychosocial causal explanations were associated with recommending medical/psychological help, while personality-related causal explanations were associated with recommending alternative help sources (e.g., family, friends or online self-help) [19]. Yet, while help-seeking intentions transferred to active help-seeking in only about a quarter of cases [20], to the best of our knowledge, the relation between different treatment recommendations and active help-seeking has not been studied in the community so far.

With regard to sociodemographic characteristics, better MHL was related to higher education and female sex [9, 21, 22], with females also being more likely to actively seek help [23, 24], already when MH problems were less severe [25]. Additionally, previous help-seeking [26] and low psychosocial functioning [25] were associated with active help-seeking, while low health satisfaction was associated with help-seeking intentions [27].

Mostly, the association between MHL and help-seeking was studied cross-sectionally, with longitudinal studies in large community samples missing. To address this gap, we investigated the impact of MHL on active help-seeking cross-sectionally as well as longitudinally in a large Swiss community sample, thereby controlling for sex, education, previous help-seeking, familiarity with MH disorders, information acquisition about health, psychosocial functioning, health satisfaction, and clinician-assessed MH problems/disorders in comprehensive models using path modelling. We expected that the correct identification of either a depression or a psychosis vignette, a biogenetic causal explanation, and recommendation of seeking help from MH professionals would be linked to active help-seeking.

## Methods

### Sample and study design

The sample consisted of participants in the baseline, add-on, and follow-up assessments of the ‘Bern Epidemiological At-Risk’ (BEAR) study, a random selection representative population telephone study in the semi-rural Canton Bern, Switzerland [28] (for further details see Online Resource sText1 and sFigures 1 and 2). Potential participants were randomly drawn from the population register. Eligibility criteria were the main residence in Canton Bern and an available telephone number. Eligible persons were first contacted by an information letter announcing first contact by telephone, and participation in the survey equaled giving informed consent. In the case of underage potential participants at baseline, the parents of these 16- and 17-year-old eligible participants were only informed about the study in an information letter that was send at the same time as the letter to the participants but not additionally asked for their consent, because according to Swiss data protection regulations, minors aged 16 and 17 years-of-age are already the sole owners of their personal data and, consequently, can agree to give information about themselves in community surveys without the additional consent of their parents. Exclusion criteria were past or present psychosis and insufficient language skills in German, French, English, or Spanish.

The total sample at baseline consisted of 2683 individuals aged 16–40 years, (response rate: 63.4%) who, between June 2011 and November 2014, were interviewed about MH problems and help-seeking. Of this cohort, 1520 German-speaking persons additionally participated in an add-on questionnaire study on MHL and stigmatization of mental disorders (response rate: 60.9%). Approximately, three years later, 1263 (47.1%) baseline participants (now at least 19 years old), preselected for a report of psychosis-risk symptoms at baseline (see Online Resource sTable 1), and age- and sex-matched controls were re-contacted between June 2015 and March 2018 and 834 completed the interview at follow-up (median follow-up: 39 months, response rate: 70.9%). Of this case–control sample, 543 (65.1%) had also participated in the baseline add-on study. Since 16 of the baseline sample and 8 of the follow-up sample had at least one missing relevant information, the final sample sizes of the path models were *N* = 1504 (cross-sectional model) and *N* = 535 (prospective model), respectively. At each stage, the BEAR study was carried out in accordance with the latest version of the Declaration of Helsinki and approved by the ethics committee of the University of Bern (No. 172/09).

### Assessments

Help-seeking at any point-of-call other than a family member or friend was assessedby the questions ‘Have you ever sought help for mental health problems?’ at baseline and ‘Have you sought help for mental health problems since the first interview?’ at follow-up. These questions are from the WHO pathway-to-care questionnaire, which has been widely used in previous studies of help-seeking behaviour [29–31]. In our study, we used a modified version with some adaptations made in terms of agencies contacted, problems presented and treatments received [32–34]. In the cross-sectional model, only help-seeking at the time of the interview was the outcome to ensure that the predictors would not have occurred after the outcome. In the prospective model, any help-seeking past baseline was the outcome.

MHL was assessed by the well-established German questionnaires of Angermeyer and colleagues [17, 25, 35–41]. The questions on causal explanations were derived from a review of the literature at the time and were further refined by the first studies using open questions [35, 36]. The MHL and stigma questionnaire of the baseline add-on study started with an unlabelled vignette on either schizophrenia or major depression, referred to in a subsequent open question on vignette identification and, also in relation to the vignette, guided questions on causal explanations, treatment recommendations and prognosis without treatment (see Online Resource sText 2 and 3).

Current symptom-independent psychosocial functioning was estimated using the Social and Occupational Functioning Assessment Scale (SOFAS) [42] with ratings ranging from 0 (poor) to 100 (superior). The SOFAS showed an excellent interrater reliability (ICC = 0.89) and high correlations with self-report measures [43].

Subjective health satisfaction was assessed with the health domain of the Brief Multidimensional Life Satisfaction Scale (BMLSS) [44] with ratings ranging from 1 (‘horrible’) to 7 (‘very satisfied’). The BMLSS revealed a high internal consistency coefficient (Cronbach’s alpha = 0.869) and good construct validity [44].

MH problems/disorders according to DSM-IV were assessed using the Mini-International Neuropsychiatric Interview (M.I.N.I.) [45]. Presence of any subthreshold MH problem that signals the need for professional assessment and, consequently, help-seeking was assumed when a screening question was affirmed [46]. The M.I.N.I. demonstrated good interrater reliability for all disorders (Kappa > 0.75) and high concordances with other structured interviews [45].

Additionally, participants were asked about targeted information acquisition about health, e.g., in the internet, newspapers, television reports or from colleagues and family (rated as specific information acquisition) versus non-specific, randomly coming-across information about health (rated as unspecific information acquisition). Furthermore, they were asked about their personal acquaintance with a person with MH problems (familiarity).

To ensure good quality and reliability of the clinical measures (SOFAS, M.I.N.I.), ratings were supervised and validated by the senior researchers, CM and FSL, before registering the final rating.

### Statistical analyses

First, orthogonal explorative factor analyses (EFA) with varimax rotation based on polychoric correlation matrices for causal explanations and treatment recommendations were computed to obtain independent factors. Missing values were imputed row wise by the median of the other items in the corresponding factor. Sampling adequacy for each analysis was checked by the Kaiser–Meyer–Olkin measure [47] and Bartlett’s test of sphericity [48]. Reliability of the factors were computed using Cronbach’s alpha [49] and Composite Reliability [50]. The factors were included in the path models as the mean value of their items.

Path models were computed using the diagonally weighted least squares estimator (DWLS) to estimate the model parameters; the weighted least squares mean and variance adjusted estimator (WLSMV) were used to estimate robust standard errors and a mean- and variance-adjusted test statistic [51]. Model fit was evaluated with the comparative fit index (CFI ≥ 0.95), the root-mean-square error of approximation (RMSEA ≤ 0.06) and the standardized root mean square residual (SRMR ≤ 0.08) [52, 53]. However, there is a severe problem, which limits the use of the χ2-statistic. It is sensitive to sample size and, as a statistical significance test, it nearly always rejects the model in large samples like ours [52]. Therefore, we followed Hu and Bentler’s ‘2-index presentation strategy’ [52] that suggests that a model should be regarded as well fitting, if RMSEA and SRMR indicate acceptable fit, i.e., if RMSEA and its 90% confidence intervals (CI), and SRMR are ≤ 0.06, with CI not containing 0.08, and ≤ 0.08, respectively [52]. After computing a cross-sectional model with satisfying fit indices for help-seeking at baseline, only significant and trend-level significant paths (*p* ≤ 0.10) were used to compute the prospective model at follow-up. Additionally, for the reported impact of sex, type of mental disorder and presence of mental problems on active help-seeking [14, 23, 25], we calculated sensitivity models for these factors. Statistical analyses were conducted in R using package *lavaan* for path models [54] and *sempower* for power analysis [55].

## Results

### Sample characteristics

At baseline, sociodemographic characteristics only differed to a small degree between persons with and without active help-seeking, whereas psychosocial functioning and MH problems/disorders clearly differed between them (Table 1). Psychosocial functioning and MH problems/disorders were associated negatively (Spearman’s *ρ* = –0.34). Thus, the higher psychosocial functioning was, the less likely MH problems and in particular MH disorders were present. At follow-up, differences in sex, education, familiarity with mental disorders, and previous help-seeking between persons with and without help-seeking were considerably higher (Table 1).

**Table 1:** Sociodemographic and clinical characteristics of the sample

	Total sample T0 (*n* = 1504)	No help-seeking T0 (*n* = 1455; 96.7%)	Help-seeking T0 (*n* = 49; 3.3%)	Statistics; effect size	Total sample T1 (*n* = 535)	No help-seeking T1 (*n* = 456; 85.2%)	Help-seeking T1 (*n* = 79; 14.8%)	Statistics; effect size
Sex: male,* n* (%)	714 (47.5%)	698 (48.0%)	16 (32.7%)	*χ*^*2*^(1) = 3.868, *p* = 0.041; *V* = 0.054	229 (42.8%)	211 (46.3%)	18 (22.8%)	*χ*^*2*^(1) = 14.228, *p* < 0.001; *V* =0 .168
Age, median (mean ± SD)	33 (30.79 ± 7.28)	33 (30.76 ± 7.28)	34 (31.61 ± 7.34)	*U* = 33,112, *p* = 0.396; *r* = – 0.007	32 (30.18 ± 7.46)	32 (30.22 ± 7.43)	32 (29.97 ± 7.70)	*U* = 18,167, *p* = 0.903; r = 0.056
Nationality: Swiss,* n* (%)	1444 (96.0%)	1396 (95.9%)	48 (98.0%)	*χ*^*2*^(1) = 0.114, *p* = 0.719; *V* = 0.018	520 (97.2%)	444 (97.4%)	76 (96.2%)	*χ*^*2*^(1) = 0.044, *p* = 0.474; *V* = 0.025
Education^a^,* n* (%)								
ISCED 2	42 (2.8%)	39 (2.7%)	3 (6.1%)	*χ*^*2*^(5) = 3.859, *p* = 0.418; *V* = 0.051	19 (3.6%)	10 (2.2%)	9 (11.4%)	*χ*^*2*^(5) = 16.723, *p* = 0.017; *V* = 0.177
ISCED 3	98 (6.5%)	93 (6.4%)	5 (10.2%)		39 (7.3%)	34 (7.5%)	5 (6.3%)	
ISCED 4	13 (0.9%)	13 (0.9%)	0 (0.0%)		8 (1.5%)	7 (1.5%)	1 (1.3%)	
ISCED 5	784 (52.1%)	761 (52.3%)	23 (46.9%)		268 (50.1%)	232 (50.9%)	36 (45.6%)	
ISCED 7	543 (36.1%)	526 (36.2%)	17 (34.7%)		195 (36.4%)	168 (36.8%)	27 (34.2%)	
ISCED 8	24 (1.6%)	23 (1.6%)	1 (2.0%)		6 (1.1%)	5 (1.1%)	1 (1.3%)	
Employment: yes,* n* (%)	1480 (98.4%)	1434 (98.6%)	46 (93.9%)	*χ*^*2*^(1) = 3.966, *p* = 0.041; *V* = 0.066	525 (98.1%)	450 (98.7%)	75 (94.9%)	*χ*^*2*^(1) = 3.315, *p* = 0.046; *V* = 0.098
Marital status,* n* (%)								
Unmarried	790 (52.6%)	762 (52.4%)	28 (57.1%)	*χ*^*2*^(2) = 4.767, *p* = 0.084; *V* = 0.056	293 (55.0%)	249 (54.8%)	44 (55.7%)	*χ*^*2*^(2) = 7.359, *p* = 0.035; *V* = 0.118
Married or registered partnership	662 (44.1%)	645 (44.4%)	17 (34.7%)		228 (42.8%)	198 (43.6%)	30 (38.0%)	
Separated/Divorced/Widowed	50 (3.3%)	46 (3.2%)	4 (8.2%)		12 (2.3%)	7 (1.5%)	5 (6.3%)	
Specific information acquisition about health: yes,* n* (%)	584 (38.8%)	560 (38.5%)	24 (49.0%)	*χ*^*2*^(1) = 1.777, *p* = 0.140; *V* = 0.038	214 (40.0%)	178 (39.0%)	36 (45.6%)	*χ*^*2*^(1) = 0.941, *p* = 0.320; *V* = 0.047
Familiarity with mental disorders: yes,* n* (%)	652 (43.4%)	622 (42.7%)	30 (61.2%)	*χ*^*2*^(1) = 5.858, *p* = .012; *V* = .066	246 (46.0%)	193 (42.3%)	53 (67.1%)	*χ*^*2*^(1) = 15.643, *p* < .001; *V* = .176
Number of previous help-seeking contacts for mental problems, median (mean ± SD)	0 (0.35 ± 0.73)	0 (0.32 ± 0.69)	1 (1.22 ± 1.30)	*U* = 18,760, *p* < 0.001; r = – 0.193	0 (0.46 ± 0.88)	0 (0.32 ± 0.70)	1 (1.24 ± 1.32)	*U* = 9672, *p* < 0.001; r = –0.353
Correct vignette identification: yes,* n* (%)	850 (56.5%)	817 (56.2%)	33 (67.3%)	*χ*^*2*^(1) = 1.984, *p* = 0.143; *V* = 0.040	319 (59.6%)	262 (57.5%)	57 (72.2%)	*χ*^*2*^(1) = 5.446, *p* < .018; *V* = .106
Prognosis without treatment (1 = progressing to 5 = completely remitting), median (mean ± SD)	1 (1.34 ± 0.74)	1 (1.34 ± 0.74)	1 (1.43 ± 0.71)	*U* = 32,645, *p* < .166; r = -0.025	1 (1.31 ± 0.71)	1 (1.32 ± 0.72)	1 (1.25 ± 0.71)	*U* = 19,160, *p* = .193; r = -0.037
Causal explanation: yes,* n* (%)								
Childhood-trauma	523 (34.8%)	501 (34.4%)	22 (44.9%)	*χ*^*2*^(1) = 1.851, *p* = 0.169; *V* = 0.039	182 (34.0%)	149 (32.7%)	33 (41.8%)	*χ*^*2*^(1) = 2.094, *p* = 0.124; *V* = 0.068
Personality	203 (13.5%)	199 (13.7%)	4 (8.2%)	*χ*^*2*^(1) = 0.807, *p* = 0.393; *V* = 0.029	75 (14.0%)	59 (12.9%)	16 (20.3%)	*χ*^*2*^(1) = 2.413, *p* = 0.112; *V* = 0.075
Psychosocial stress	1209 (80.4%)	1169 (80.3%)	40 (81.6%)	*χ*^*2*^(1) = 0.002, *p* = 0.968; *V* = 0.006	434 (81.1%)	373 (81.8%)	61 (77.2%)	*χ*^*2*^(1) = 0.649, *p* = 0.351; *V* = 0.042
Substance abuse	931 (61.9%)	904 (62.1%)	27 (55.1%)	*χ*^*2*^(1) = 0.717, *p* = 0.370; *V* = 0.026	328 (61.3%)	279 (61.2%)	49 (62.0%)	*χ*^*2*^(1) = 0.001, *p* = 0.987; *V* = 0.006
Biogenetics	816 (54.3%)	792 (54.4%)	24 (49.0%)	*χ*^*2*^(1) = 0.370, *p* = 0.470; *V* = 0.019	292 (54.6%)	242 (53.1%)	50 (63.3%)	*χ*^*2*^(1) = 2.440, *p* = 0.111; *V* = 0.073
Treatment recommendation: yes,* n* (%)								
Alternative medicine	450 (29.9%)	434 (29.8%)	16 (32.7%)	*χ*^*2*^(1) = 0.071, *p* = 0.638; *V* = 0.011	168 (31.4%)	141 (30.9%)	27 (34.2%)	*χ*^*2*^(1) = 0.198, *p* = 0.600; *V* = 0.025
Self-care	738 (49.1%)	712 (48.9%)	26 (53.1%)	*χ*^*2*^(1) = 0.179, *p* = 0.663; *V* = 0.015	282 (52.7%)	234 (51.3%)	48 (60.8%)	*χ*^*2*^(1) = 2.045, *p* = .143; *V* = .067
Mental health professional	1301 (86.5%)	1256 (86.3%)	45 (91.8%)	*χ*^*2*^(1) = 0.807, *p* = 0.393; *V* = 0.029	457 (85.4%)	386 (84.6%)	71 (89.9%)	*χ*^*2*^(1) = 1.086, *p* = 0.299; *V* = 0.053
Health satisfaction (1 = horrible to 7 = very satisfied), median (mean ± SD)	6 (6.07 ± 0.91)	6 (6.11 ± 0.88)	5 (5.16 ± 1.16)	*U* = 52,067, *p* < 0.001; r = – 0.149	6 (6.03 ± 0.93)	6 (6.12 ± 0.86)	6 (5.52 ± 1.15)	*U* = 23,471, *p* < .001; r = –0.193
Psychosocial functioning^b^, median (mean ± SD)	87 (85.79 ± 6.35)	88 (86.17 ± 5.68)	78 (74.37 ± 12.08)	*U* = 58,883, *p* < 0.001; r = – 0.200	87 (85.41 ± 7.23)	88 (86.51 ± 5.24)	83 (79.08 ± 12.25)	*U* = 24,566, *p* < 0.001; r = – 0.220
Mental health problems^c^,* n* (%)								
No mental health problem	1009 (67.1%)	997 (68.5%)	12 (24.5%)	*χ*^*2*^(2) = 136.95, *p* < 0.001; *V* = 0.302	318 (59.4%)	288 (63.2%)	30 (38.0%)	*χ*^*2*^(2) = 55.051, *p* < 0.001; *V* = 0.321
Only mental health problem, no mental health disorder^d^	387 (25.7%)	374 (25.7%)	13 (26.5%)		163 (30.5%)	140 (30.7%)	23 (29.1%)	
Mental health disorder	108 (7.2%)	84 (5.8%)	24 (49.0%)		54 (10.1%)	28 (6.1%)	26 (32.9%)	

^**a**^International standard classification of education 2011 (No participants with ISCED 1 and ISCED 6)

^**b**^Social and occupational functioning scale (SOFAS, 0–100, lower scores indicate lower psychosocial functioning)

^**c**^Excluding specific phobia

^**d**^Rated when a screening question of the M.I.N.I. was affirmed but the full criteria were not met

### Factors of causal explanations und treatment recommendations

The Kaiser–Meyer–Olkin measure verified the sampling adequacy for each analysis, KMO = 0.798 (causal explanations) and KMO = 0.778 (help recommendations), which are both evaluated as ‘middling’ according to Kaiser (1979). All KMO values for individual items were > 0.681 (causal explanations) and > 0.557 (help recommendations), which are both above the acceptable limit of 0.500 [47]. Bartlett’s test of sphericity, *χ*^2^(153) = 6061.48, *p* < 0.001 (causal explanations) and *χ*^2^(136) = 6034.86, *p* < 0.001 (help recommendations), indicated that correlations between items were sufficiently large for the EFA [48]. Thus, the sample and data were adequate to run a factor analysis. The overall factor solution explained 42% (help recommendations) and 46% of the variance (causal explanations) and both the Kaiser’s criterion and the scree plot (see Online Resource sFigures 3 and 4) converged on the respective number of factors. Thus, five factors for the causal explanations and four factors for the help recommendations were retained in the final analysis. Orthogonal EFA with varimax rotation of the 18 causal explanations revealed the following five independent factors: ‘childhood trauma’, ‘substance abuse’, ‘psychosocial stress’, ‘personality’, and ‘biogenetics’ (Table 2). Orthogonal EFA with varimax rotation of the 17 treatment recommendations revealed the following four independent factors: ‘MH professionals’, ‘alternative medicine’, ‘self-care’, and ‘other’ (Table 3). The factor ‘other’ demonstrated only low factor loadings (< 0.50) and, consequently, was not included in the path models. Scale reliabilities of the factors were in an acceptable to good range (Table 2 and 3).

**Table 2:** Results of the explorative factor analysis of the 18 causal explanations

Items	Factor 1: psychosocial stress	Factor 2: substance abuse	Factor 3: childhood trauma	Factor 4: personality	Factor 5: biogenetics	Communality
Burdens and worries in partnership and family	**0.67**	0.07	0.16	0.09	– 0.17	0.52
Too demanding of oneself (too ambitious, too strict with oneself)	**0.62**	– 0.01	0.04	0.11	– 0.02	0.40
Shock from a drastic life event such as the loss of someone close to you	**0.51**	0.24	0.19	0.05	0.00	0.36
General hustle and bustle of contemporary life	**0.63**	– 0.10	0.11	0.16	– 0.02	0.44
Job stress and worries (including unemployment)	**0.84**	0.02	0.10	0.05	– 0.05	0.72
An unconscious conflict	**0.38**	– 0.04	0.09	0.13	0.06	0.17
Abuse of medication or drugs	– 0.04	**0.84**	0.07	0.13	0.18	0.75
Excessive consumption of alcohol	0.10	**0.75**	0.11	0.15	0.16	0.63
Growing up in a broken family or in an institution	0.08	0.16	**0.75**	0.10	– 0.01	0.60
Unkind treatment at home or too strict upbringing	0.27	0.09	**0.73**	0.17	0.09	0.65
Lack of support from other people	0.25	-0.01	**0.31**	0.16	– 0.19	0.22
Spoiling or overprotective parents	0.14	0.00	**0.41**	0.30	0.03	0.28
Weak constitution (has always been low in resilience, very sensitive or nervous	0.18	0.06	0.05	**0.51**	0.14	0.31
Immoral lifestyle	0.09	0.26	0.18	**0.53**	– 0.15	0.42
Weakness of will	0.16	0.06	0.08	**0.70**	– 0.20	0.57
God's will	0.04	0.02	0.15	**0.38**	0.03	0.17
Heredity	0.04	0.16	– 0.01	0.00	**0.78**	0.64
Disease of the brain	– 0.23	0.32	0.07	–0.07	**0.47**	0.39
Eigenvalue	2.63	1.57	1.53	1.46	1.06	
Cronbachs‘ alpha	0.80	0.82	0.69	0.64	0.60	
Composite reliability	0.77	0.77	0.67	0.59	0.56	

Bold values represent the highest loading of the corresponding item on the factors

**Table 3:** Results of the explorative factor analysis of the 17 treatment recommendations

Items	Factor 1: mental health professionals	Factor 2: alternative medicine	Factor 3: self-care	Factor 4: other	Communality
Psychotherapist	**0.79**	0.06	0.07	0.04	0.63
Social psychiatric service	**0.59**	– 0.08	– 0.01	0.12	0.37
Psychiatrist	**0.81**	– 0.08	– 0.05	– 0.09	0.68
Psychotherapy	**0.81**	0.01	– 0.03	– 0.02	0.65
Medications for the mind (psychotropic drugs)	**0.51**	– 0.16	– 0.13	0.04	0.30
Naturopath or other alternative doctor	– 0.04	**0.74**	0.21	0.17	0.62
Natural remedies	– 0.12	**0.86**	0.15	0.20	0.82
Acupuncture	– 0.10	**0.72**	0.34	0.15	0.67
Concentration or relaxation exercises (autogenic training)	– 0.06	0.31	**0.77**	0.10	0.71
Meditation or yoga	– 0.07	0.32	**0.75**	0.11	0.69
Self-help group	0.11	0.08	0.16	**0.36**	0.17
Family doctor or general practitioner	0.13	0.04	– 0.02	**0.26**	0.09
Cure	– 0.05	0.15	0.15	**0.42**	0.22
Educational counselor or other counseling service	0.07	0.04	0.15	**0.45**	0.23
Pastor/priest	– 0.04	– 0.01	– 0.09	**0.41**	0.18
Trusted person	– 0.13	0.04	0.14	**0.30**	0.13
Electroconvulsive treatment (therapy with electric shocks)	0.00	0.08	– 0.04	**0.22**	0.06
Eigenvalue	2.63	2.09	1.48	1.02	
Cronbachs ‘ alpha	0.83	0.87	0.82	0.50	
Composite reliability	0.78	0.84	0.79	0.44	

Bold values represent the highest loading of the corresponding item on the factors

### Cross-sectional model

**Fig. 1:**
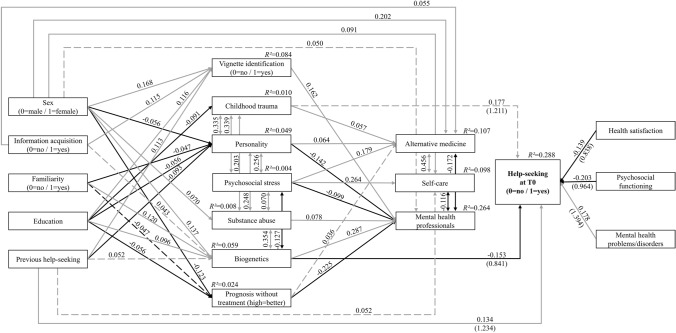
Cross-sectional model (*N* = 1504) with standardized path coefficients**.** Model fit indices: *χ*^*2*^(41) = 109.687 with *p* < 0.001, CFI = 0.962, SRMR = 0.028, RMSEA = 0.033 (90%CI = 0.026–0.041). Power > 0.999. Odds ratios in brackets for the endogenous variable Help-seeking at T0. Solid lines indicate significant paths (*p* ≤ 0.05), dashed lines indicate marginally significant paths (*p* ≤ 0.10), grey indicates positive associations, black indicates negative associations

The cross-sectional model showed excellent fit and power, both its trend-level significant and significant paths (*p* ≤ 0.10) are displayed in Fig. 1 (see Online Resource sTable 2 for standardized regressions and covariance values). At significance level (*p* ≤ 0.05), of all 18 modelled associations, help-seeking at baseline was merely directly positively associated with previous help-seeking and the presence of MH problems/disorders, and negatively associated with biogenetic causal explanations, psychosocial functioning, and health satisfaction. No associations between help-seeking at baseline and treatment recommendations were found. Professional treatment recommendations were positively associated with the causal explanations ‘biogenetics’ and ‘substance abuse’, the correct identification of the vignette, and the assumption of a poor prognosis without treatment, and negatively associated with the causal explanations ‘personality’ and ‘psychosocial stress’. Furthermore, sex and targeted information acquisition were directly related only to treatment recommendations but not help-seeking, and familiarity and education were related to causal explanations but not help-seeking. Female sex, targeted information acquisition, higher education and previous help-seeking were associated with correct identification of the vignette as a proxy measure of a good MHL, which, however, was not related to help-seeking.

### Prospective model

**Fig. 2:**
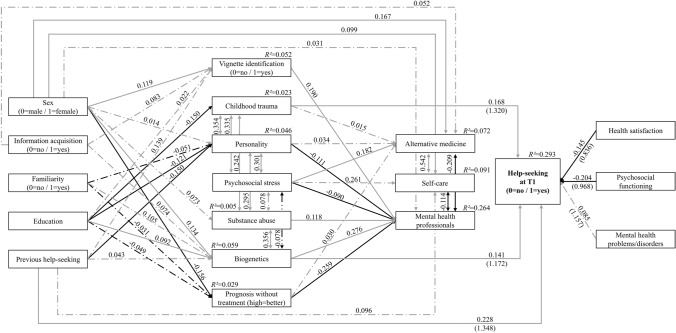
Prospective model (*N* = 535) with standardized path coefficients. Model fit indices: *χ*^*2*^(92) = 124.810 with *p* = 0.013, CFI = 0.954, SRMR = 0.053, RMSEA = 0.026 (90%CI = 0.012–0.037). Power > 0.999. Odds ratios in brackets for the endogenous variable Help-seeking at T1. Solid lines indicate significant paths (*p* ≤ 0.05), dashed and dotted lines indicate non-significant paths (*p* > 0.05), grey indicates positive associations, black indicates negative associations

The prospective model that was based on the (trend-level) significant paths of the cross-sectional model (*p*  ≤  0.10; Fig. 1) also showed an excellent fit and power (Fig. 2; see Online Resource sTable 2 for standardized regressions and covariance values). At the significance level, help-seeking at follow-up remained positively influenced by the previous help-seeking, and also remained negatively influenced by psychosocial functioning and health satisfaction. The presence of baseline MH problems/disorders, however, lost its significant effect on help-seeking. Newly, the causal explanation ‘childhood trauma’ had a significant positive effect on help-seeking. The effect of the causal explanation ‘biogenetics’ remained significant but was reversed, i.e., the effect of ‘biogenetics’ on help-seeking was positive in the cross-sectional model and negative in the prospective model. The other paths were largely in the same direction as in the cross-sectional model, although fewer reached the level of significance.

### Mediation effect

In the prospective path model, two mediations from education via the causal explanations ‘childhood trauma’ and ‘biogenetics’, respectively, to help-seeking at follow-up were indicated and, thus, tested for a mediating effect of the respective causal explanation. However, both mediation analyses (see Online Resource sFigure 3) revealed no significant indirect effect supporting mediation.

### Sensitivity analyses of the prospective model

The sensitivity analyses revealed models of good fit (see Online Resource sFigures 4–9), except for males and participants with MH problems/disorders, likely due to reduced sample size and power.

Positive effects of the causal explanations ‘childhood trauma’ and ‘biogenetics’ on help seeking at follow-up were found only in males, participants with the depression vignette, and participants with MH problems/disorders. Interestingly, help-seeking at follow-up was influenced by psychosocial functioning only in males, participants with the schizophrenia vignette, and participants with MH problems/disorders, while it was influenced by health satisfaction only in females, participants with the depression vignette and participants without MH problems/disorders.

## Discussion

In this first-time study of both cross-sectional and longitudinal predictors of help-seeking for mental problems/disorders, we examined the relationship between relevant aspects of MHL, sociodemographic predictors of help-seeking, and active help-seeking in a Swiss community sample of young adults. Our expectation that MHL, in terms of correct identification of an unlabelled vignette (schizophrenia or depression), a biogenetic causal explanation, and recommendation of seeking help from MH professionals, would be linked to subsequent active help-seeking was only partially supported for biogenetic causal explanations. In addition, endorsements of the causal explanation ‘childhood trauma’, and, in line with previous studies [25–27], previous help-seeking, lower psychosocial functioning, and lower health satisfaction were also associated with subsequent help-seeking. Furthermore, sensitivity analyses revealed a significant impact of sex, type of vignette, and presence of MH problems/disorders, although these variables were not associated with help-seeking in the overall prospective model.

### Comparison of the cross-sectional and the prospective model

Interestingly, and in line with reports from cross-sectional studies [10, 56, 57], the expected positive association of biogenetic causal explanations with help-seeking was replicated in our prospective model but was reversed in the cross-sectional model in that the causal explanation ‘biogenetics’ was negatively associated with help-seeking. However, previous studies, including earlier analyses of this sample [12], reported an association between biogenetic causal explanations and negative stereotypes, and between negative stereotypes and a wish for social distance, which is commonly negatively associated with active help-seeking [12, 38, 58–61]. Thus, it could be assumed that the fear of stigma linked to biogenetic causal explanations because of associated negative stereotypes might have prevented active help-seeking initially. Yet, these explanations may have facilitated help-seeking in the long run when, possibly, fear of identifying with the negative stereotypes related to biogenetic causal explanations increased in terms of evolving self-stigma. Regardless, future studies will have to examine these potential and unclear relationships between biogenetic causal explanation and emerging self-stigma [62–64] as well as help-seeking. Another interesting difference between the cross-sectional and prospective model was the role of MH problems/disorders that were significantly associated with help-seeking only at baseline. One reason might be a change in MH state, in particular remission of symptoms, that had weakened the relationship between baseline MH problems/disorders and help-seeking at follow-up. However, at baseline, only 7.5% of persons with MH problems/disorders had sought help, while this number increased to 22.6% at follow-up. Another reason might be the new occurrence of MH problems/disorders up until follow-up. This is supported by the declining number of persons with baseline MH problems/disorders amongst help-seekers at baseline (75.5%) and follow-up (62.0%). Thus, future longitudinal studies should include MH problems/disorders at any assessment time to compare their long-term and immediate effects on help-seeking.

Contrary to previous studies [8, 9], in both models, a correct identification of the vignette was not associated with help-seeking. Since a significant association between correct labelling and help-seeking was also found in our data at follow-up (but not at baseline) when only the two variables were considered, the stronger association of causal explanations likely outperformed that of correct labelling.

A similarly unexpected finding was that both models demonstrated no associations between treatment recommendations and help-seeking. This might be due to operationalizing treatment recommendations into three categories, while the point of help-seeking was not similarly differentiated but broadly included several types of institutions, ranging from school, church and police via primary care and counselling services to MH services. Thus, likely specific associations between specific treatment recommendations and actual points of contact would not have shown up here for the single outcome ‘help-seeking’. Future studies of larger sample size or oversampled for persons with help-seeking could help to investigate these links in more detail.

### Group-specific results on the role of health satisfaction and functioning

When the role of MH problems/disorders was further analysed using sensitivity analyses, clear differences emerged. While low health satisfaction was the sole predictor for help-seeking in persons without MH problems/disorders, in those with MH problems/disorders, help-seeking was predicted by lower psychosocial functioning, previous help-seeking, and the causal explanations ‘childhood trauma’ and ‘biogenetics’. Yet, for the lower number of participants with MH problems/disorders, the latter model was slightly underpowered and would need to be re-examined in larger samples of sufficient power. The differences between these two models might be explained in terms of the nature of MH problems/disorders and the point of help-seeking contact. Health satisfaction was assessed with a general question and not specifically with regard to MH. Thus, the MH problems of the group without positive screening answers to the M.I.N.I. might have been mostly emotional distress in response to intrapersonal, interpersonal, or role performance stressors or somatic health problems that would have not been considered psychopathological symptoms in the interview [65]. Therefore, emotional stress might have mostly influenced health satisfaction rather than psychosocial functioning that consequently, was significantly associated with the number of MH problems/disorders [25] and, in our sample, was more strongly correlated with MH problems/disorders (Spearman’s *ρ* = –0.34) compared to health satisfaction (Spearman’s *ρ* = –0.24).

Similar to the model on persons with MH problems/disorders, significant effects of functioning and the causal explanations ‘biogenetics’ and ‘childhood trauma’ were also found in males but not in females, in whom, similar to the model of persons without MH problems/disorders, health satisfaction and baseline help-seeking predicted subsequent help-seeking. These similarities in the two types of sensitivity models appeared to be independent of each other because baseline MH problems/disorders did not significantly differ in the follow-up sample between females (43.1%) compared to males (37.1%; *χ*^*2*^(1) = 1.727, *p* = 0.182, Cramer’s *V* = 0.061). The sex differences in the role of emotional (health satisfaction) and functional (psychosocial functioning) triggers for help-seeking, are in line with reports of men focusing more on problem-solving and females more on emotional distress when seeking help [66–69].

### Group-specific results on the role of the previous help-seeking

The role of the previous help-seeking also differed in the sensitivity analyses, playing a significant role in females and, independent of sex, in participants with MH problems/disorders but not in males and those without MH problems/disorders. While the result in the sensitivity models according to the presence of MH problems/disorders likely reflects that a need for care had not newly occurred past baseline, the results in the sex-specific models likely reflect the fact that the MH treatment gap is larger in males [23, 24]. This had already been reported for the baseline assessment of the BEAR study, with only 34.2% of males with MH problems/disorders compared to 50.7% of females having reported help-seeking [25]. Furthermore, persons seeking help for emotional distress not captured by the M.I.N.I. screening questions, e.g. in relation to intrapersonal, interpersonal or role performance problems [65], might be more likely to seek help from their personal network rather than official institutions [70–72]. Thus, as our points of help-seeking contacts only included several types of institutions, they were less named by participants without MH problems/disorders compared to those with MH problems/disorders.

For the broad inclusion of institutions, however, it would be interesting to study a possible gradient from help-seeking from friends/relatives in case of emotional distress via help-seeking from primary and semi-professional care services in case of MH problems to help-seeking from professional MH services in case of MH disorders [71]. Understanding factors that influence help-seeking at different severity levels of MH problems would increase the general understanding of help-seeking and, relatedly, barriers to it. Unfortunately, for the already low number of persons with/without MH problems and, in particular, disorders, we could not study this gradient due to statistical power reasons.

### Group-specific results on the role of the causal explanations ‘biogenetics’ and ‘childhood trauma’

The causal explanations ‘childhood trauma’ and ‘biogenetics’ positively influenced help-seeking only in males and in participants with MH problems/disorders. Furthermore, they were positively associated with subsequent help-seeking only in participants who had been presented with the depression vignette. In line with our overall model, an impact of sex on the relationship between causal explanations and help-seeking was not reported in previous studies when sex entered as a predictor [10, 38, 56, 57]. Yet, the results of our sensitivity analyses indicate that males are more likely to base their decision for help-seeking on their own causal explanation when these involve factors out of their current control. This would be in line with studies reporting a strong tendency for males to try to deal with their problems themselves [73]. Females, however, might be more likely seek help depending on their level of distress regardless of their own causal explanations [67, 69]. This might be another reason why females more often seek help than males [23, 24]. Furthermore, causal explanations might be more relevant to participants with MH problems/disorders compared to those suffering from emotional distress only, in relation to an already identified stressor, such as intra- or interpersonal, or role performance stressors. The exact relationship between the severity of MH problems and the importance of causal explanations in the decision to seek help needs to be explored in future studies.

The difference between the two types of vignettes might be related to the nature of MH problems/disorders for that help was sought, and their resemblance with the case vignette. Depression is the most common mental disorder [1, 74] and depressive mood is one of the main reasons for help-seeking [23, 75], and this was also found at the baseline of the BEAR study [76]. Baseline depressive problems/disorders were also frequent in help-seekers at follow-up (39.2%), whereby interviews were terminated when a diagnosis of a psychotic disorder was assured. Thus, almost every second participant with MH problems/disorder could identify with the depression but likely only few with the schizophrenia vignette and, consequently, the recommendations, beliefs and help-seeking intentions stated for the depression vignette were more likely reflecting real-life considerations underlying actual help-seeking behaviour. Therefore, the causal explanations for the depression vignette were likely more systematically related to help-seeking than the causal explanations for the schizophrenia vignette, thus resulting in significant paths and the highest explained variance of help-seeking in the subgroup with the depression vignette (*R*^*2*^ = 0.451) compared to all other models (*R*^*2*^ = 0.143–0.293).

Commonly, a schizophrenia vignette has been more strongly related to biogenetic causal explanations compared to a depression vignette, which commonly has been mostly related to psychosocial causal explanations [12, 17, 18]. Childhood trauma, however, was given as a causal explanation for several mental disorders, including alcohol abuse, where it was specifically associated with recommendation of drug treatment [77]. Interestingly, despite the relevance in our models and the growing empirical evidence [78], childhood trauma has been increasingly less regarded as a cause of mental disorders in Germany between 1990 and 2011 [17]. The difference between our findings, and the relationships between type of disorder depicted in a vignette, and main causal explanation described in the literature irrespective of their association with help-seeking [12, 17, 18] indicates that the relation between causal explanations and help-seeking may be problem-specific. Thus, general disorder- or problem-unspecific models might fail to apply to certain groups of persons or give conflicting results in different groups, such as the apparent contradictory association of the biogenetic causal explanation in the cross-sectional and prospective model. Future community-based studies on the role of causal explanations should therefore include a wider range of vignettes and consider the MH status of the participant to be able to match participants’ problems to the vignette to generate problem-specific models of help-seeking.

### Strengths and limitations

This study has several clear strengths: active help-seeking behaviour as the outcome (rather than only help-seeking intentions), the prospective design, and the sufficiently large sample size that allowed consideration of complex overall path models with excellent power. Nevertheless, more than 95% of the sample consisted of Swiss people between 16 and 40 years of age at baseline, so that the results can only be generalized to young and middle-aged adults in Western cultures. The factors ‘Biogenetics,’ ‘Substance abuse,’ and ‘Self-care’ consist of only two items, which is below the recommended minimum number of four variables of a factor [79]. Furthermore, in particular due to the comparably low loading and explained variance (communality) of the causal explanation ‘Disease of the brain’, the factor ‘Biogenetics’ just reached the minimal acceptable internal consistence and eigenvalue [49, 79], while these indicated that ‘Substance abuse,’ and ‘Self-care’ were consistent and important factors despite including only two items whose variance was well explained by the factors (communalities  ≥  0.63). However, because of the construct immanence and meaningfulness of these two-item factors as well as their reported importance in MHL and help-seeking, in particular of ‘Biogenetics’ [11, 12, 17, 18, 38, 62], we decided to include these factors in our models. The very good fit of our models might be regarded as indicative of saturated models and, consequently, of over-optimistic model evaluations due to their complex nature. A saturated model would be one, in which the number of free parameters exactly equals the number of known values, i.e., a model with zero degrees of freedom [80]. Yet, in our models the number of known values has always been greater than the number of estimated parameters so that we had a positive number of degrees of freedom to conduct fit tests. In addition, two of the sensitivity analyses were slightly underpowered due to the small subsample size of males (*n* = 229) and participants with MH problems/disorders (*n* = 217). This led to empirical underidentification in these two models, with a non-reliable estimation of parameters, as indicated by negative variances [53, 80, 81]. Thus, interpretation of these two models needs to be done with some caution.

## Conclusion and Implications

 Overall, our prospective model highlighted the role of the causal explanations ‘biogenetics’ and ‘childhood trauma’, previous help-seeking, health satisfaction, and psychosocial functioning in the decision to actively seek help in a Swiss community sample. However, our sensitivity analyses revealed that these associations played a different role in various subgroups that might be relevant in the development of group-specific programs to advance early help-seeking and advertise early treatments. For example, such programs might focus more on actual functional problems in males, while highlighting health satisfaction in females. Thus, future studies on promotors of and barriers to help-seeking should assess very large community samples with case vignettes on different mental disorders to examine appropriate subgroups and their likely interaction to address group-specific factors in awareness campaigns.

## Supplementary Information

Below is the link to the electronic supplementary material.

Supplementary file1 (PDF 2630 KB)

## Data Availability

Data are available upon reasonable request from the senior author at frauke.schultze-lutter@lvr.de. Participants of the BEAR study gave informed consent to sharing anonymized data.
