# Supplementary material for: Influence of mental health literacy on help-seeking behaviour for mental health problems in the Swiss young adult community: a cohort and longitudinal case–control study
Source: Eur Arch Psychiatry Clin Neurosci. 2022 Sep 11;273(3):649–62. doi: 10.1007/s00406-022-01483-9 (PMC10085901; doi:10.1007/s00406-022-01483-9)
Supplement: Supplementary file 1 — Supplementary file1 (PDF 2630 KB) [file 406_2022_1483_MOESM1_ESM.pdf]

Supplementary Information

Influence of mental health literacy on help-seeking behaviour for mental health problems in the Swiss young adult community: a cohort and longitudinal case-control study.

Journal: European Archives of Psychiatry and Clinical Neuroscience

N. Osman[1], C. Michel[2], B. G. Schimmelmann[2,3], L. Schilbach[1,4], E. Meisenzahl[1] and F. Schultze-Lutter[1,2,5]

[1]Department of Psychiatry and Psychotherapy/LVR-Klinikum Düsseldorf, Medical Faculty, Heinrich-Heine-University Germany

[2]University Hospital of Child and Adolescent Psychiatry and Psychotherapy, University of Bern, Switzerland

[3]University Hospital of Child and Adolescent Psychiatry, University Hospital Hamburg-Eppendorf, Hamburg, Germany

[4]Medical Faculty, Ludwig Maximilians Universität, München, Germany

[5]Department of Psychology, Faculty of Psychology, Airlangga University, Indonesia

Corresponding author: Naweed Osman, MSc, Department of Psychiatry and Psychotherapy/LVR-Klinikum Düsseldorf, Medical Faculty, Heinrich-Heine University Düsseldorf, Bergische Landstraße 2, 40629 Düsseldorf, Germany. naweed.osman@lvr.de. ORCID: 0000-0001-9761-0099.

Content

sTable 1. Clinical high risk for psychosis (CHR) criteria. **3**

sFigure 1. Results of recruitment until interview at baseline. **5**

sFigure 2. Results of recruitment after interview at baseline. **6**

sText 1. Further details on study design, recruitment and sample. **7**

sText 2. English translation of the original German vignettes. **10**

sText 3. Further explanations of the assessments of vignette identification, causal explanations, treatment recommendations and prognosis without treatment. **11**

sFigure 3. Scree plot of the explorative factor analysis of the 18 causal explanations. **12**

sFigure 4. Scree plot of the explorative factor analysis of the 17 treatment recommendations. **13**

sFigure 5. Multiple mediation analysis for prospective model (*N*=535), with standardized path coefficients **14**

sFigure 6. Prospective model, only males (*n*=229), with standardized path coefficients **18**

sFigure 7. Prospective model, only females (*n*=306), with standardized path coefficients. **19**

sFigure 8. Prospective model, only schizophrenia vignette (*n*=281), with standardized path coefficients. **20**

sFigure 9. Prospective model, only depression vignette (*n*=254), with standardized path coefficients. **21**

sFigure 10. Prospective model, only participants without mental health problems or disorders (*n*=318), with standardized path coefficients. **22**

sFigure 11. Prospective model, only participants with mental health problems or disorders (*n*=217), with standardized path coefficients. **23**

References **24**

sTable 1. Clinical high risk for psychosis (CHR) criteria.

**Ultra-high risk (UHR) criteria as per the Structured Interview for Psychosis-Risk Syndromes (SIPS; [1])**
Brief Intermittent Psychotic Symptoms Syndrome (**BIPSS**)<br>≥1 of the following SIPS positive (P) items scored 6 = 'severe and psychotic' with first appearance in the past 3 months and a presence of at least several minutes per day at a frequency of at least once per month but less than 7 days:<br>P1 Unusual Thought Content / Delusional Ideas<br>P2 Suspiciousness / Persecutory Ideas<br>P3 Grandiose Ideas<br>P4 Perceptual Abnormalities / Hallucinations<br>P5 Disorganized Communication
Attenuated Psychotic Symptoms Syndrome (**APSS**)<br>≥1 of the above P-items scored 3 = 'moderate' to 5 = 'severe but not psychotic' with a first appearance within the past year or current rating one or more scale points higher compared to 12 months ago, and with an occurrence of an average frequency of at least once per week in the past month.
Genetic Risk and Deterioration Syndrome (**GRDS**)<br>Patient meets SIPS-criteria for Schizotypal Personality Disorder or has 1st-degree relative with a psychotic disorder, and has experienced >30% drop in the global assessment of functioning score over the last month compared to 12 months ago
**Basic symptom (BS) criteria as per the Schizophrenia Proneness Instruments (SPI; [2, 3])**
Cognitive-Perceptive Basic Symptoms (**COPER**)<br>≥1 of the following BS with a score of ≥3 = 'frequency of at least several times in a month or weekly' within the last 3 months and with first occurrence ≥12 months ago:<br>Thought interference<br>Thought perseveration<br>Thought pressure<br>Thought blockages<br>Disturbance of receptive speech<br>Decreased ability to discriminate between ideas/perception, fantasy/memories<br>Unstable ideas of reference<br>Derealization

Visual perception disturbances (excl. hypersensitivity to light or blurred vision)<br>Acoustic perception disturbances (excl. hypersensitivity to sounds)
Cognitive Disturbances (**COGDIS**)<br>≥2 of the following BS with a score of ≥3 within the last 3 months:<br>Inability to divide attention<br>Thought interference<br>Thought pressure<br>Thought blockages<br>Disturbance of receptive speech<br>Disturbance of expressive speech<br>Unstable ideas of reference<br>Disturbances of abstract thinking<br>Captivation of attention by details of the visual field

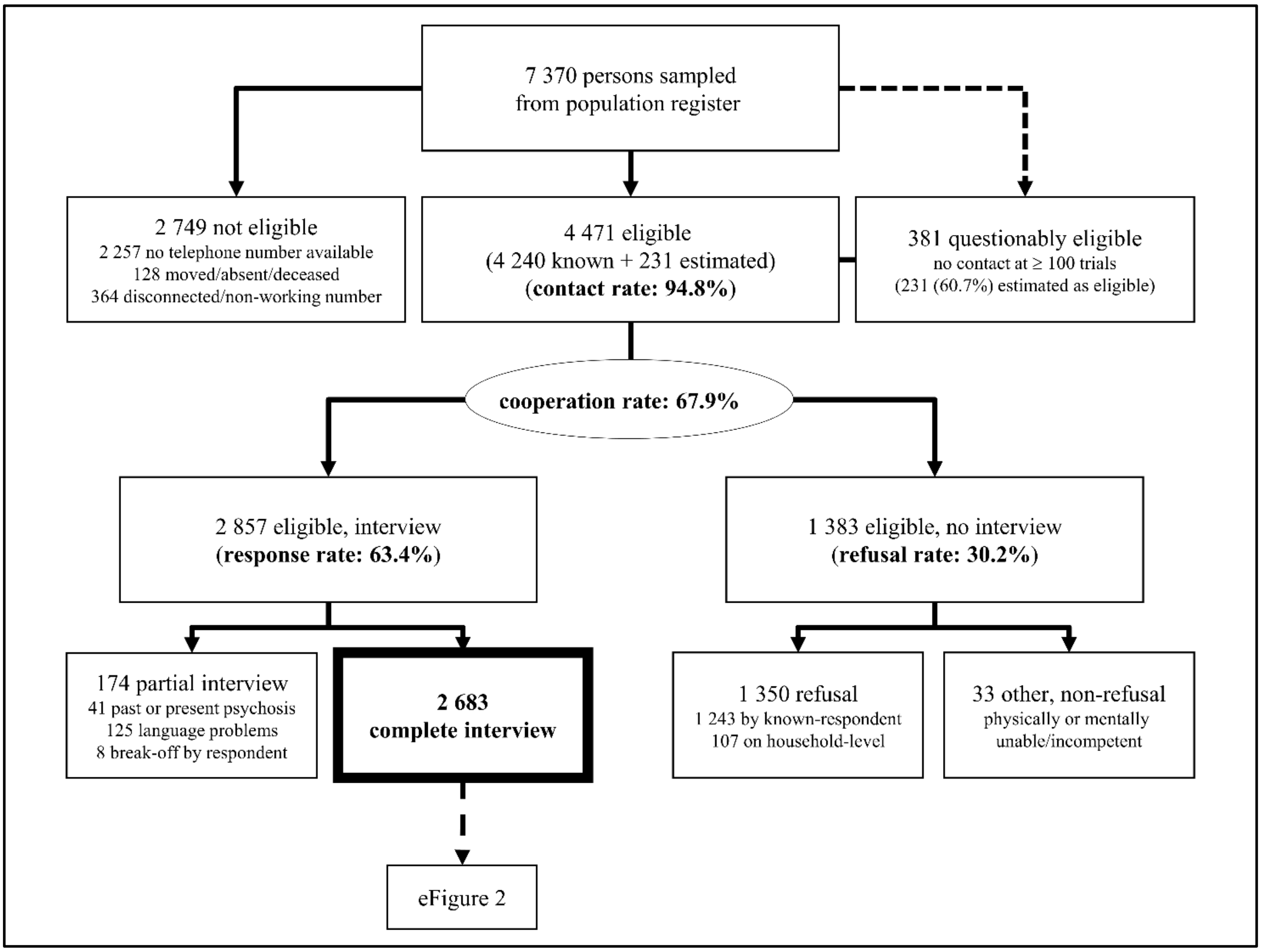

sFigure 1. Results of recruitment until interview at baseline.

Survey outcome rates of the BEAR study according to the definitions of the American Association for Public Opinion Research [4].

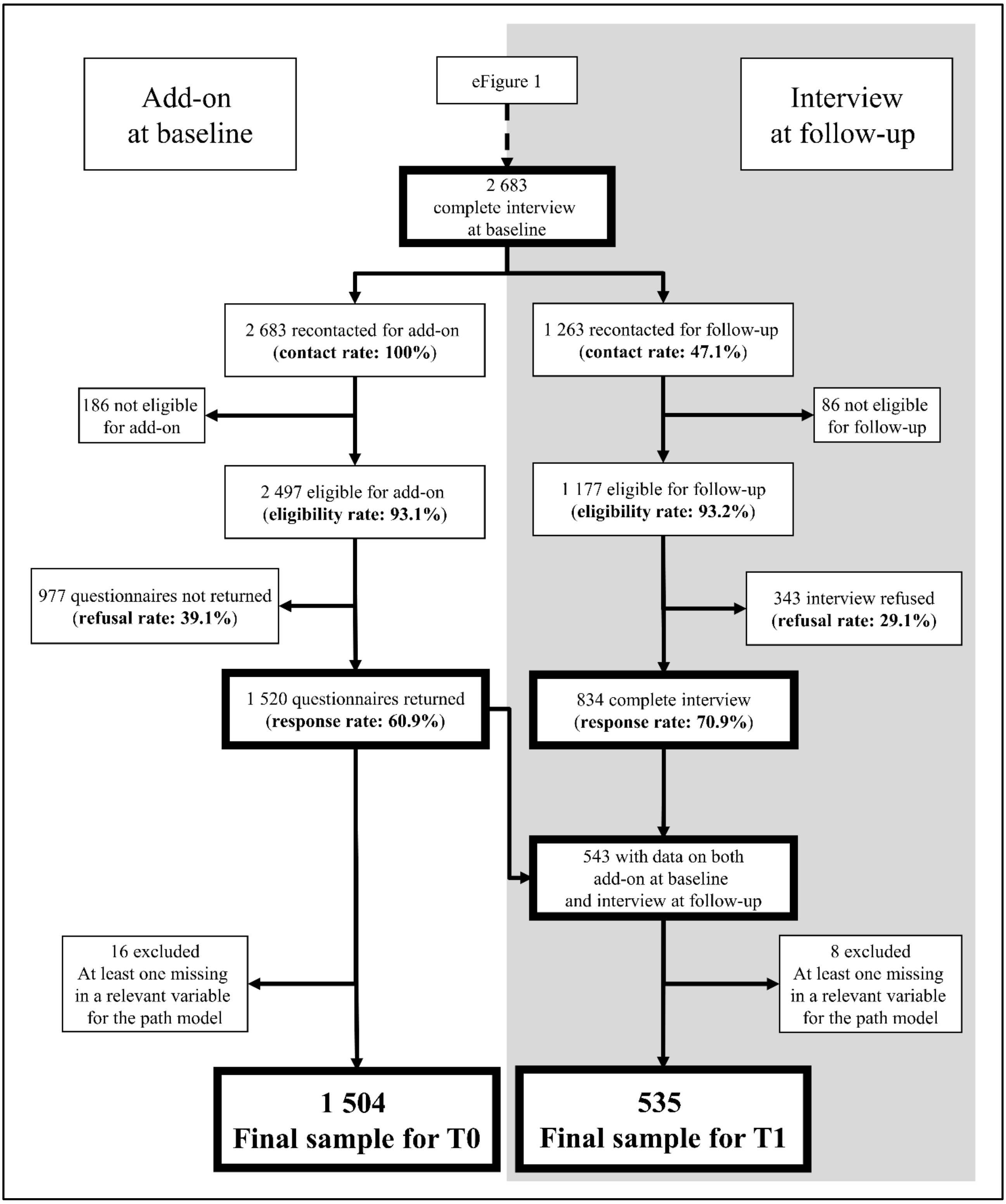

sFigure 2. Results of recruitment after interview at baseline.

Survey outcome rates of the BEAR study according to the definitions of the American Association for Public Opinion Research [4].

sText 1. Further details on study design, recruitment and sample.

Details on study design

To increase response rates, contact was initially established using a one-page information letter at both baseline and follow-up. First telephone contact was attempted within two weeks of sending the letter. After detailed explanation of goals and proceedings of the study, participation in the telephone interview was considered as giving informed consent.

At baseline, inclusion criteria were being of eligible age (16-40 years) and being a main resident of Canton Bern (i.e. having a valid address and not being abroad during the assessment period). At follow-up, inclusion criteria were participation in baseline interview and consent to be re-contacted. In addition. an available telephone number was required for eligibility at both baseline and follow-up. We called participants up to 100 times over several months at various times and days, including Saturdays. Potential participants that were not reached within this time were considered as unknown eligible. Moreover, interviews at both baseline and follow-up were aborted prematurely when respondents had (i) a lifetime diagnosis of psychosis1 or, at baseline, (ii) insufficient language skills in German, French, or English. The semi-structured interviews lasted 43 minutes on average (SD: 20 minutes; range: 20–225 minutes) at baseline, and 52 minutes on average (SD: 26 minutes; range: 24–248 minutes) at follow-up. The BEAR study was carried out in accordance with the latest version of the Declaration of Helsinki. Further details on baseline recruitment and sample are provided in Schultze-Lutter et al. (2018, 2021).

Details on recruitment of sample and representativeness

Baseline assessment

At baseline, from the 4471 eligible participants, 2857 interviews were conducted [5]. However, 125 (4.4%) of the 2857 interviews were aborted prematurely by the interviewer for insufficient language skills; 41 (1.4%) were aborted for a lifetime diagnosis of psychosis, which had not been diagnosed or treated in 19 cases [6]; and 8 (0.3%) were terminated prematurely by the participants themselves. Lack of time or interest was the main reason given by the 1350 (29.5%) refusers. Thus, according to the definitions of the American Association for Public Opinion Research [4], the contact rate was 94.8%, and the response rate, 63.4% with 2683 completed interviews [5] (see sFigure 1).

The eligible sample was negligibly older than the 16- to 40-year-old general population of Bern, mainly because a non-significant higher number of available telephone numbers (landlines) was found for 36- to 40-year-olds [5]. Similar to the observation for the eligibility sample, the 2683 interviewees differed negligibly, i.e., at less than small effect size, from the 16- to 40-year-old general population of Bern in age distribution, but not in sex, nationality, and marital status. Consequently,

as no response bias was detectable beyond the extremely small age-related inclusion bias, the interviewees were regarded as representative of their age group [5].

Of the 2683 interviewees, 2539 were sufficiently fluent in German to be eligible for the add-on questionnaire study of mental health literacy and stigma. Of these, 324 refused to participate. Of the 2215 interviewees who agreed to the add-on study, 689 did not returned the questionnaire after a maximum of three reminder calls, while 1520 returned the questionnaire. Thus, according to the definitions of the American Association for Public Opinion Research [4], the contact rate of the add-on study was 72.9%, the cooperation rate 82.5%, the refusal rate 12.8%, and the response rate 60.9% [5] (see sFigure 2).

Follow-up assessment

Originally, the follow-up recruitment period was planned to span over 36 months. However, due to the PI (FSL) leaving Switzerland, funding stopped slightly earlier, and the study was concluded two months earlier. Thus, during the abbreviated 34 months of recruitment (June 2015-March 2018), the original recruitment aims of n=500 persons who had reported at least one lifetime clinical high risk (CHR) symptom (RISK) at baseline and n=500 matched persons who had not reported any CHR symptom (CONTROL) were not fully reached, and the contact rate was only 78.8% [7]. Furthermore, until conclusion of the study, contact with the target person could only be re-established in 995 persons, resulting in a cooperation rate of 84.7%. Of these 995 persons, 151 refused to participate again in the study (see sFigure 2). The main reasons for refusal given by the n=86 (57.0%) refusers who agreed to participate in a non-responder interview were similar to those given by refusers of the baseline [5]. In descending order and with multiple answers possible, reasons for refusal at follow-up were: 46 (53.5%) lack of time, 37 (43.0%) lack of interest, 11 (12.8%) interview too long, 10 (11.6%) too intimate and/or private questions, 6 (7.0%) no personal gain, 3 (3.5%) irrelevant topic. Of the 834 interviewees with a full interview [5], 434 had been sampled as RISK subjects (52.0%) and 400 as CONTROL subjects (48.0%). Thus, slightly more sampled RISK than sampled CONTROL had participated until early study termination ($\chi^2(1)=6.832$, p=0.009, Cramer's V=0.074). However, interviewed RISK and CONTROL did not differ in baseline age (RISK: 30.4±7.7 yrs., Mdn=32.2 yrs.; CONTROL: 30.2±7.7 yrs., Mdn=32.4 yrs.; U=85384.5, p= 0.684, Rosenthal's r=0.014), sex (RISK: 46.5% male, CONTROL: 47.3% male; $\chi^2(1)=0.042$, p=0.838, Cramer's V=0.007), baseline nationality (RISK: 96.3% Swiss, CONTROL: 96.3% Swiss; $\chi^2(1)=0.002$, p=0.961, Cramer's V=0.002), baseline highest educational level (RISK: 86.3% ISCED 5 or higher (International Standard Classification of Education 2011), CONTROL: 86.1% ISCED 5 or higher; $\chi^2(1)=4.549$, p=0.715, Cramer's V=0.074), and baseline partnership (RISK: 56.9% single, CONTROL: 56.8% single; $\chi^2(1)=0.002$,

p=0.962, Cramer’s V=0.002). Thus, despite the pre-term conclusion of the study and the negligible bias towards recruitment of RISK, matching was sufficient and both samples were well comparable [7]. Of the 834 participants with a complete follow-up interview, 543 (65.1%) had participated in the add-on study at baseline and returned the questionnaire (see sFigure 2).

sText 2. English translation of the original German vignettes.

Imagine that you know the following about an acquaintance (AB) with whom you occasionally spend your leisure time.

Depression vignette, unlabelled:

Within the past 2 months, AB has changed in nature. In contrast to previously, AB is down and sad without being able to give a concrete reason for feeling low. AB appears serious and worried. There is no longer anything that will make AB laugh. AB hardly ever talks, and if AB says something, AB speaks in a low tone of voice about the worries AB has with regard to AB’s future. AB feels useless and has the impression AB does everything wrong. All attempts to cheer AB up have failed. AB lost all interest in things and is not motivated to do anything. AB complains of often waking up in the middle of the night and not being able to get back to sleep. By the morning, AB feels exhausted and without energy. AB says that AB encounters difficulty in concentrating on AB’s job. Unlike before, everything takes AB a very long time to do. AB hardly manages AB’s workload. As a consequence, AB has already been summoned to AB’s boss. AB has now sought professional help and was told AB appears to be suffering from depression.

Psychosis vignette, unlabelled:

In the past months, AB appears to have changed. More and more, AB has retreated from their friends and colleagues, up to the point of avoiding them. If someone managed to involve AB in a conversation, AB would only talk about whether some people have the natural gift of reading other people’s thoughts. This question became AB’s sole concern. In contrast with AB’s previous habits, AB has stopped taking care of their appearance and looked increasingly untidy. At work, AB seemed absent-minded and frequently made mistakes. As a consequence, AB has already been summoned to their boss. Finally, AB stayed away from work for an entire week without an excuse. Upon their return, AB seemed anxious and harassed. AB now reports being absolutely certain that people cannot only read other people’s thoughts but also directly influence them. AB was, however, unsure who would steer ABs thoughts. AB also said that, when thinking, AB was continually interrupted. Frequently, AB would even hear those people talk to AB, and they would give AB instructions. Sometimes, they would also talk to each other and make fun of whatever AB was doing at the time. AB said that the situation was particularly bad at AB’s apartment. At home, AB would really feel threatened, and would be terribly scared. Hence, AB had not spent the night at AB’s place for the past week, but rather had hidden in hotel rooms and hardly dared to go out. AB has now sought professional help and was told AB appears to be suffering from schizophrenia.

sText 3. Further explanations of the assessments of vignette identification, causal explanations, treatment recommendations and prognosis without treatment.

For assessment of vignette identification, participants were asked in an open question ‘Please write down in key words what you would call the condition this person is in? What do you think this person has?’. The answer in the participants' own words was then evaluated and coded into 0=‘correct psychiatric diagnosis’ or 1=‘incorrect psychiatric diagnosis’ [8]. Examples of a correct labelling of the depression vignette were statements like affective or mood disorder, depression, depressive, or depressed; and of the schizophrenia vignette like psychosis, psychotic, psychotic disorder, paranoia, paranoid, schizophrenia, hallucination, or delusion. Examples of statements rated as incorrect are mental or psychiatric disorder, bipolar, anxiety or personality disorder, over-burdened, burn-out, having a personal or mental problem, sadness, sad, low mood, mood swings, midlife or life crisis, stress, out of touch with reality, madness, mad, disoriented, or confused [8]. For assessment of causal explanation, participants were asked "Now we would like to know what you think can cause such a problem as you have read. For your answer you have an answer scale with 5 points. For each possibility, please mark the extent to which it could be the cause of such a problem.". Participants then had to rate 18 causes (see Table 2) on a five-point Likert scale from 1=‘certainly not a cause’ to 5=‘certainly a cause’. For assessment of treatment recommendation, participants were asked "Nowadays, there are a variety of help options to solve such a problem. Some possibilities are listed below. For each option, please mark with a cross the extent to which you would recommend or advise against using it to solve the problem described. Given a problem like this, would you advise ...". Participants then had to rate 10 institutions and 7 treatments (see Table 3) on a five-point Likert scale from 1=‘would strongly advise’ to 5=‘would strongly advise against it’ or alternatively 88=‘I cannot say’, which would represent a missing value. For assessment of progression prognosis, participants were asked to predict the progression without treatment on a five-point scale from 0=‘Your problem will probably disappear completely and never occur again’ to 4=‘Your problem will probably get worse and worse’ or alternatively 88=‘I cannot say’, which would represent a missing value.

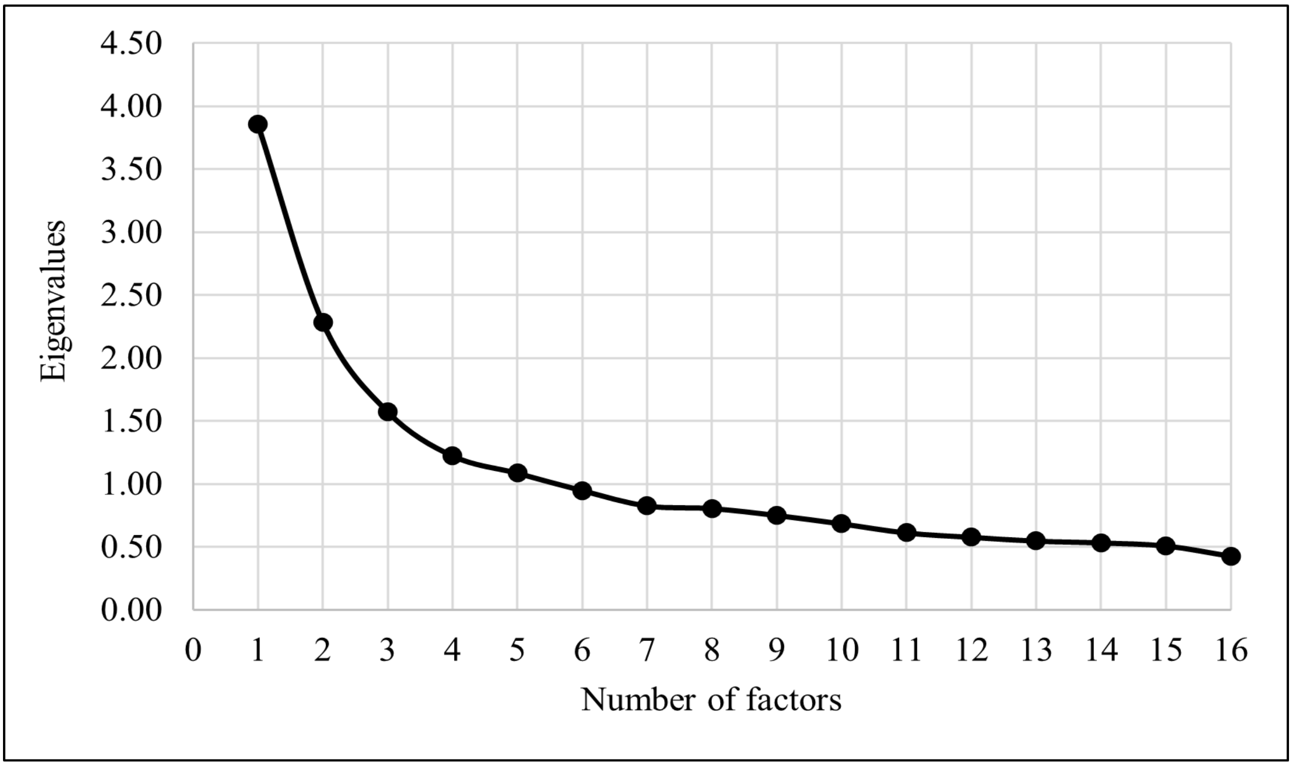

sFigure 3. Scree plot of the explorative factor analysis of the 18 causal explanations.

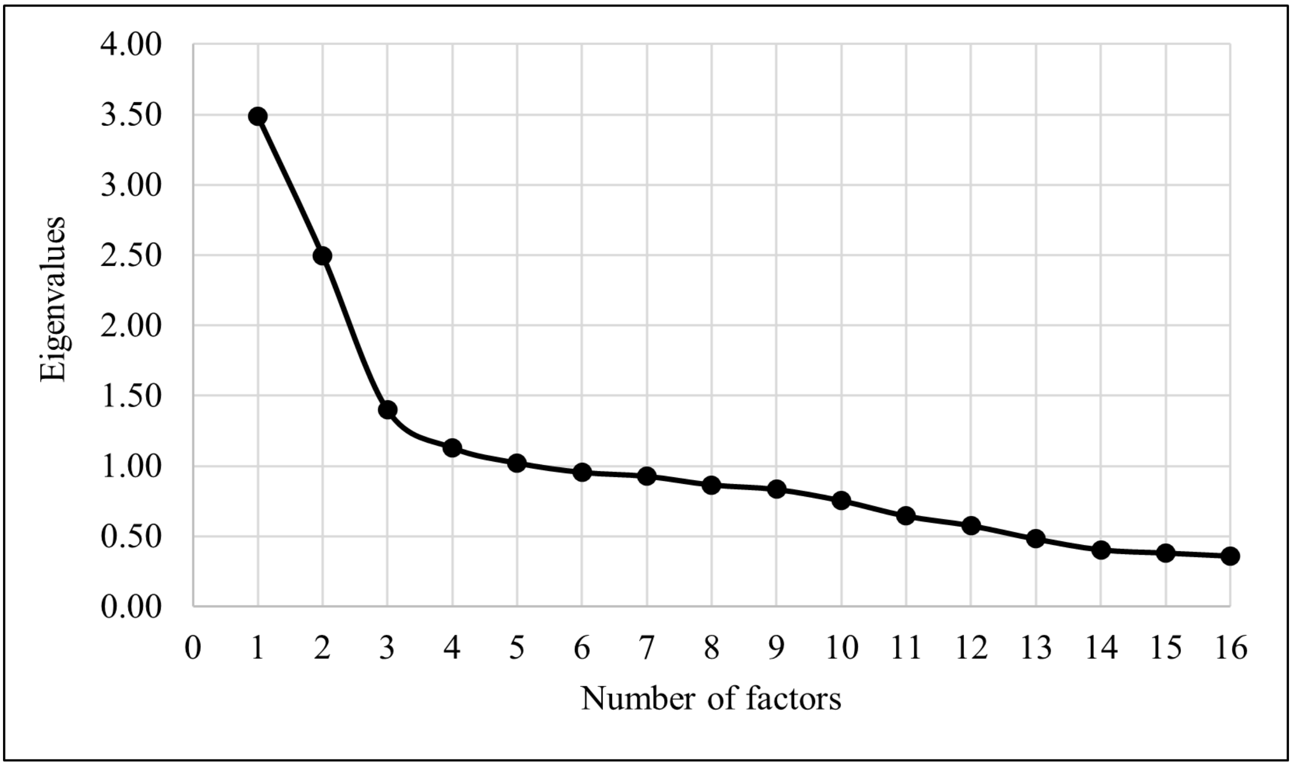

sFigure 4. Scree plot of the explorative factor analysis of the 17 treatment recommendations.

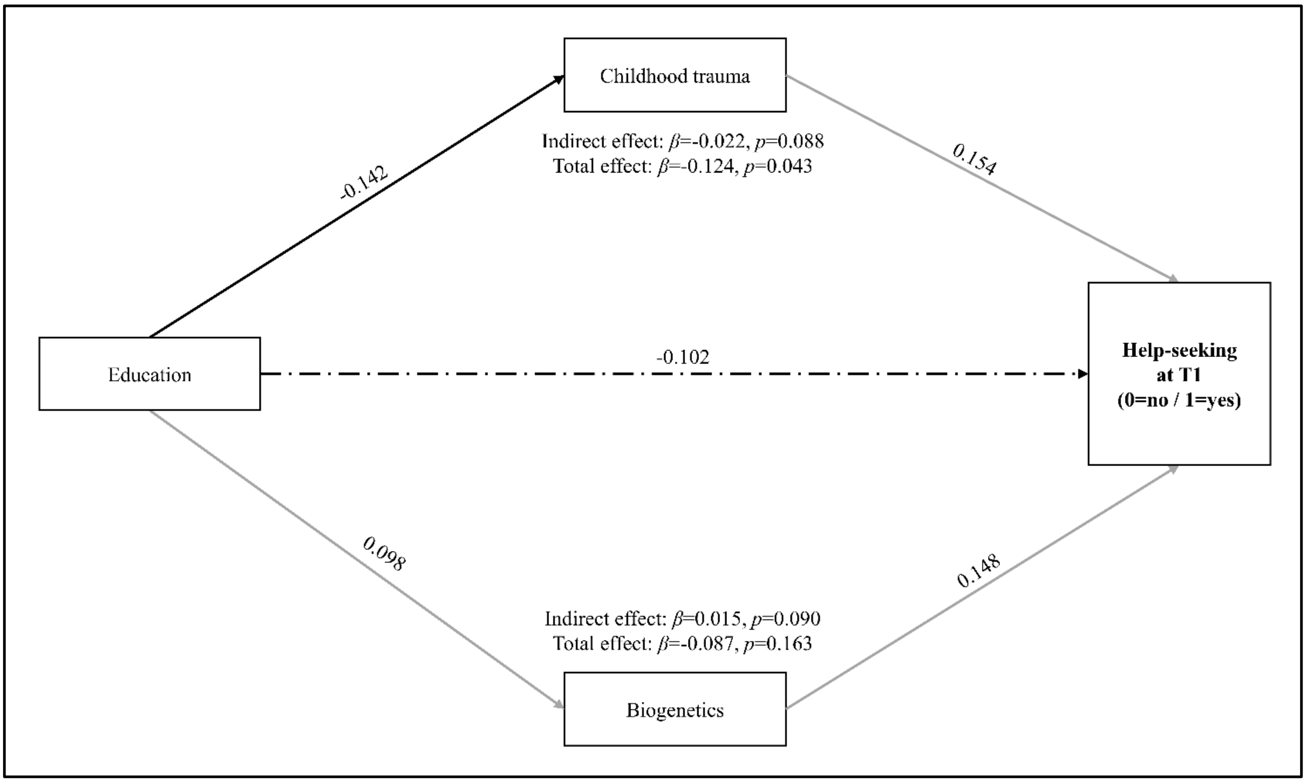

sFigure 5. Multiple mediation analysis for prospective model (N=535), with standardized path coefficients.

Solid lines indicate significant paths ($p \leq 0.05$), dashed and dotted lines indicate non-significant paths ($p > 0.05$), grey indicates positive associations, black indicates negative associations.

sTable 2. Standardized regressions and covariances of both models.

	**Standardized Beta (Odds Ratio) in Cross-Sectional Model (*n*=1504)**	**Standardized Beta (Odds Ratio) in Prospective Model (*n*=535)**
**Help-seeking ~**		
Vignette identification	0.077 (1.089)	
Psychosocial stress	-0.065 (0.895)	
Substance abuse	-0.034 (0.960)	
Childhood trauma	0.117* (1.211)	0.168** (1.320)
Personality	-0.019 (0.968)	
Biogenetics	-0.153** (0.841)	0.141** (1.172)
Mental health professionals	0.097 (1.159)	
Alternative medicine	-0.016 (0.982)	
Self-help	-0.046 (0.948)	
Prognosis without treatment	0.031 (1.050)	
Sex	0.111 (1.293)	
Education	-0.009 (0.992)	
Previous help-seeking	0.134** (1.234)	0.228** (1.348)
Familiarity	-0.013 (0.969)	
Information acquisition	0.055 (1.140)	
Mental health problems	0.178** (1.394)	0.085 (1.157)
Psychosocial functioning	-0.203** (0.964)	-0.204** (0.968)
Health satisfaction	-0.139** (0.838)	-0.145** (0.836)
**Vignette identification ~**		
Sex	0.168**	0.119**
Education	0.116**	0.022
Previous help-seeking	0.113**	0.139
Familiarity	0.035	
Information acquisition	0.115**	0.083
**Psychosocial stress ~**		
Sex	0.031	
Education	-0.025	
Previous help-seeking	0.033	
Familiarity	-0.035	
Information acquisition	0.014	
**Substance abuse ~**		
Sex	0.070**	0.073
Education	-0.014	
Previous help-seeking	0.030	
Familiarity	-0.008	
Information acquisition	0.030	
**Childhood trauma ~**		
Sex	-0.042	
Education	-0.091**	-0.150**
Previous help-seeking	-0.017	
Familiarity	-0.010	

	**Standardized Beta (Odds Ratio) in Cross-Sectional Model (*n*=1504)**	**Standardized Beta (Odds Ratio) in Prospective Model (*n*=535)**
Information acquisition	0.019	
**Personality ~**		
Sex	-0.056**	0.014
Education	-0.168**	-0.121**
Previous help-seeking	-0.093**	-0.150**
Familiarity	-0.061**	-0.051
Information acquisition	0.007	
**Biogenetics ~**		
Sex	0.137**	0.134**
Education	0.096**	0.092**
Previous help-seeking	0.052*	0.043
Familiarity	0.120**	0.105**
Information acquisition	0.043*	0.074
**Prognosis without treatment ~**		
Sex	-0.123**	-0.156**
Education	-0.056**	-0.049
Previous help-seeking	-0.025	
Familiarity	-0.047*	-0.031
Information acquisition	-0.002	
**Mental health professionals ~**		
Sex	0.050*	0.031
Education	0.005	
Previous help-seeking	0.045	
Familiarity	0.004	
Information acquisition	-0.013	
Vignette identification	0.162**	0.190**
Psychosocial stress	-0.099**	-0.090**
Substance abuse	0.078**	0.118**
Childhood trauma	0.009	
Personality	-0.142**	-0.111**
Biogenetics	0.287**	0.276**
Prognosis without treatment	-0.225**	-0.259**
**Alternative medicine ~**		
Sex	0.202**	0.167**
Education	-0.018	
Previous help-seeking	0.044	
Familiarity	0.025	
Information acquisition	0.055**	0.052
Vignette identification	-0.042	
Psychosocial stress	0.179**	0.182**
Substance abuse	-0.010	
Childhood trauma	0.057**	0.015
Personality	0.064**	0.034

	**Standardized Beta (Odds Ratio) in Cross-Sectional Model (*n*=1504)**	**Standardized Beta (Odds Ratio) in Prospective Model (*n*=535)**
Biogenetics	-0.038	
Prognosis without treatment	0.036*	0.030
**Self-care ~**		
Sex	0.091**	0.099**
Education	0.011	
Previous help-seeking	0.052*	0.096
Familiarity	0.022	
Information acquisition	0.026	
Vignette identification	0.010	
Psychosocial stress	0.264**	0.261**
Substance abuse	0.028	
Childhood trauma	0.011	
Personality	0.026	
Biogenetics	-0.029	
Prognosis without treatment	0.043	
**Psychosocial stress ~~**		
Substance abuse	0.070**	0.078
Childhood trauma	0.339**	0.335**
Personality	0.256**	0.301**
Biogenetics	-0.127**	-0.078
**Substance abuse ~~**		
Childhood trauma	0.203**	0.242**
Personality	0.248**	0.295**
Biogenetics	0.354**	0.356**
**Childhood trauma ~~**		
Personality	0.335**	0.354**
Biogenetics	0.021	
**Personality ~~**		
Biogenetics	0.005	
**Mental health professionals ~~**		
Alternative medicine	-0.172**	-0.209**
Self-help	-0.116**	-0.114**
**Alternative medicine ~~**		
Self-help	0.456**	0.542**

* $p \leq 0.10$, ** $p \leq 0.05$, ~ predicted by, ~~ correlated with

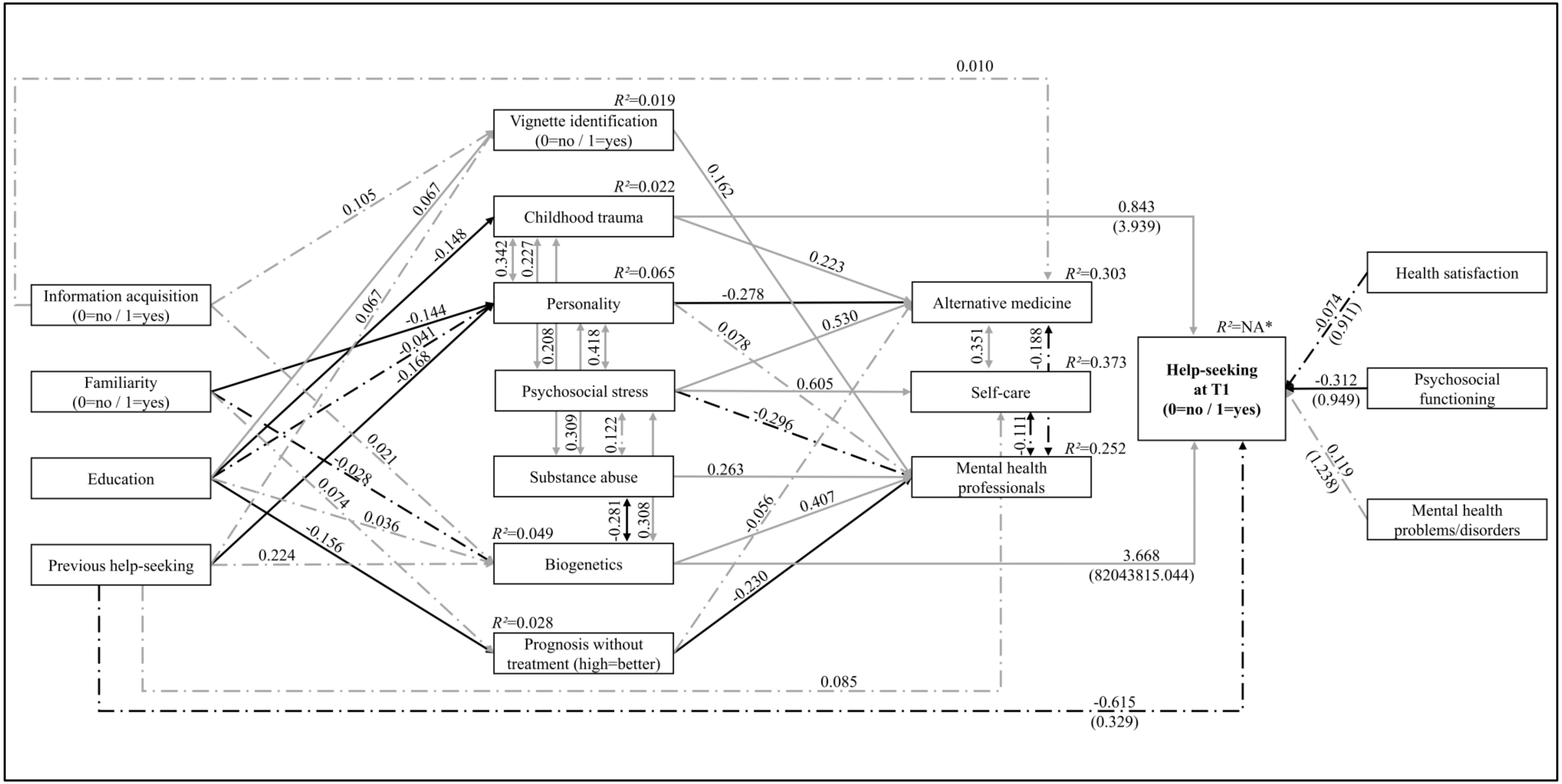

sFigure 6. Prospective model, only males (*n*=229), with standardized path coefficients.

Model fit indices: $\chi 2(89)=272.904$ with $p<0.001$, CFI=0.928, SRMR=0.162, RMSEA=0.095 (90%CI=0.082-0.108). Power=0.929.

Odds ratios in brackets for the endogenous variable Help-seeking at T1. Solid lines indicate significant paths ($p \leq 0.05$), dashed and dotted lines indicate non-significant paths ($p>0.05$), grey indicates positive associations, black indicates negative associations. *R² not available because of empirical underidentification indicated by negative error variance.

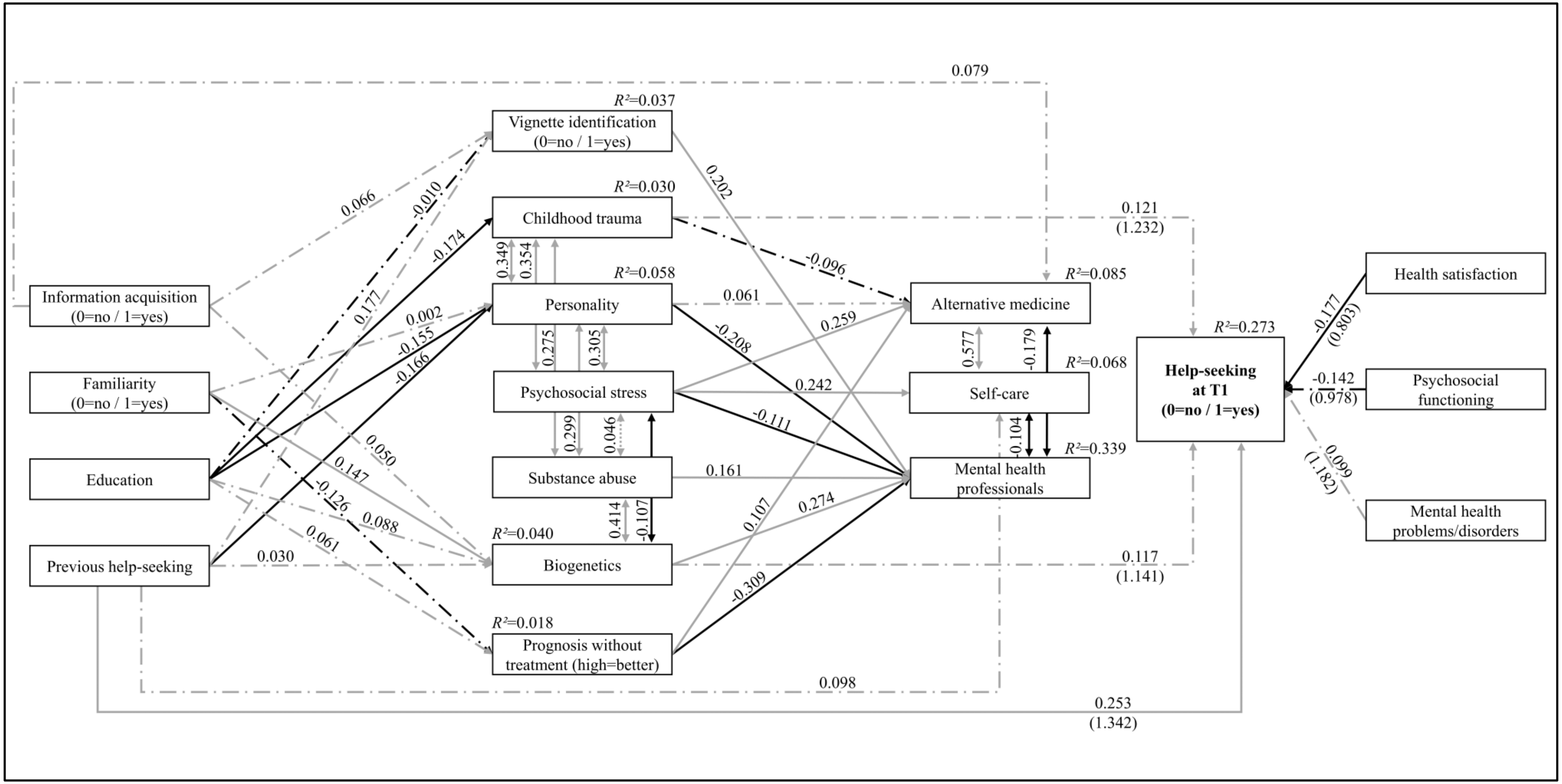

sFigure 7. Prospective model, only females (n=306), with standardized path coefficients.

Model fit indices: $\chi 2(89)=108.217$ with $p=0.081$, CFI=0.960, SRMR=0.055, RMSEA=0.027 (90%CI=0.000-0.043). Power=0.989.

Odds ratios in brackets for the endogenous variable Help-seeking at T1. Solid lines indicate significant paths ($p \leq 0.05$), dashed and dotted lines indicate non-significant paths ($p>0.05$), grey indicates positive associations, black indicates negative associations.

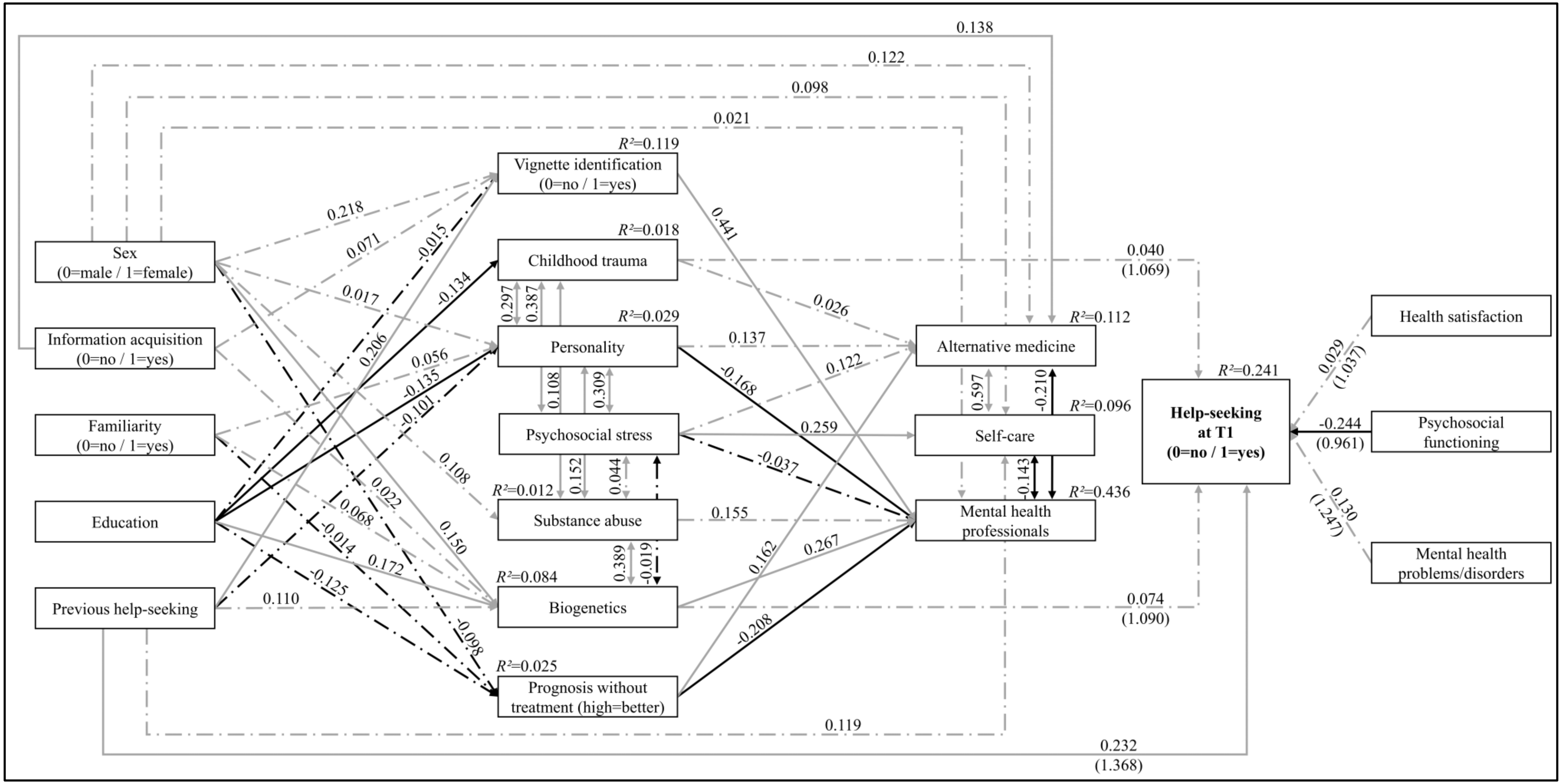

sFigure 8. Prospective model, only schizophrenia vignette (n=281), with standardized path coefficients.

Model fit indices: $\chi 2$(92)=123.079 with p=0.017, CFI=0.937, SRMR=0.074, RMSEA=0.035 (90%CI=0.015-0.050). Power=0.981.

Odds ratios in brackets for the endogenous variable Help-seeking at T1. Solid lines indicate significant paths ($p \leq 0.05$), dashed and dotted lines indicate non-significant paths ($p > 0.05$), grey indicates positive associations, black indicates negative associations.

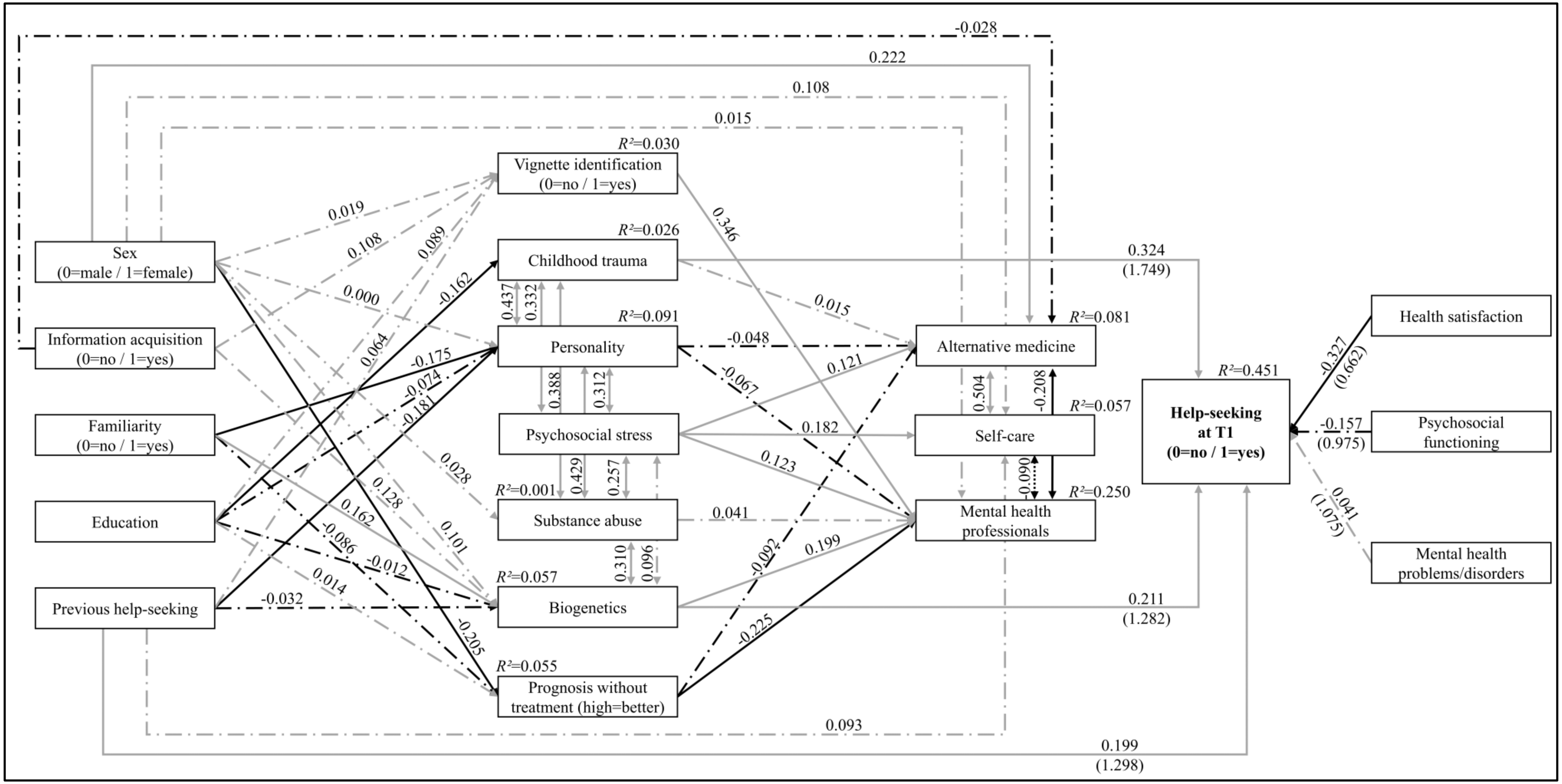

sFigure 9. Prospective model, only depression vignette (n=254), with standardized path coefficients.

Model fit indices: $\chi 2(92)=121.536$ with $p=0.021$, CFI=0.922, SRMR=0.074, RMSEA=0.036 (90%CI=0.015-0.052). Power=0.964.

Odds ratios in brackets for the endogenous variable Help-seeking at T1. Solid lines indicate significant paths ($p \leq 0.05$), dashed and dotted lines indicate non-significant paths ($p > 0.05$), grey indicates positive associations, black indicates negative associations.

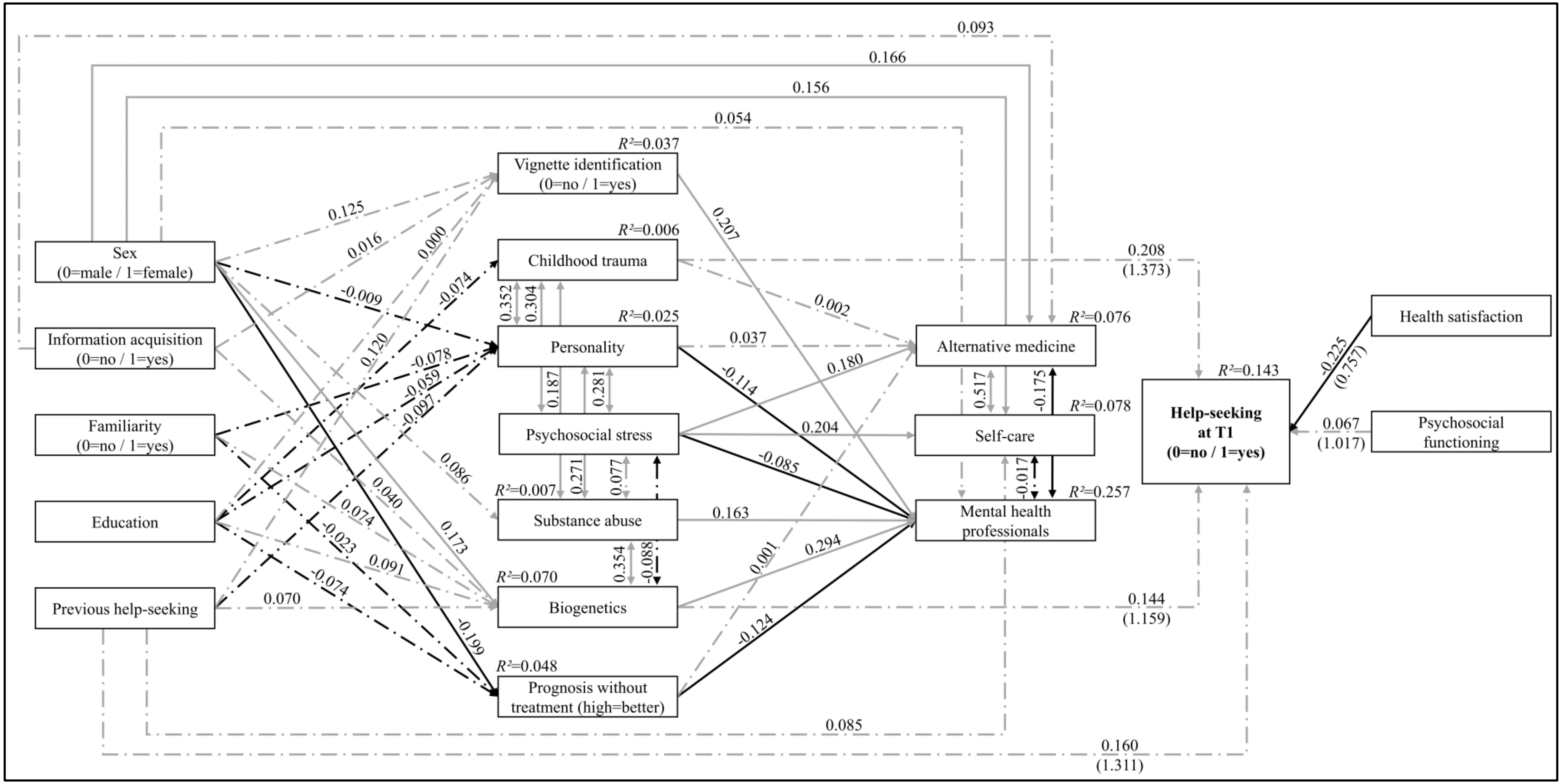

sFigure 10. Prospective model, only participants without mental health problems or disorders (n=318), with standardized path coefficients.

Model fit indices: $\chi 2$(82)=86.346 with p=0.350, CFI=0.987, SRMR=0.051, RMSEA=0.013 (90%CI=0.000-0.034). Power=0.988.

Odds ratios in brackets for the endogenous variable Help-seeking at T1. Solid lines indicate significant paths ($p \leq 0.05$), dashed and dotted lines indicate non-significant paths (p>0.05), grey indicates positive associations, black indicates negative associations.

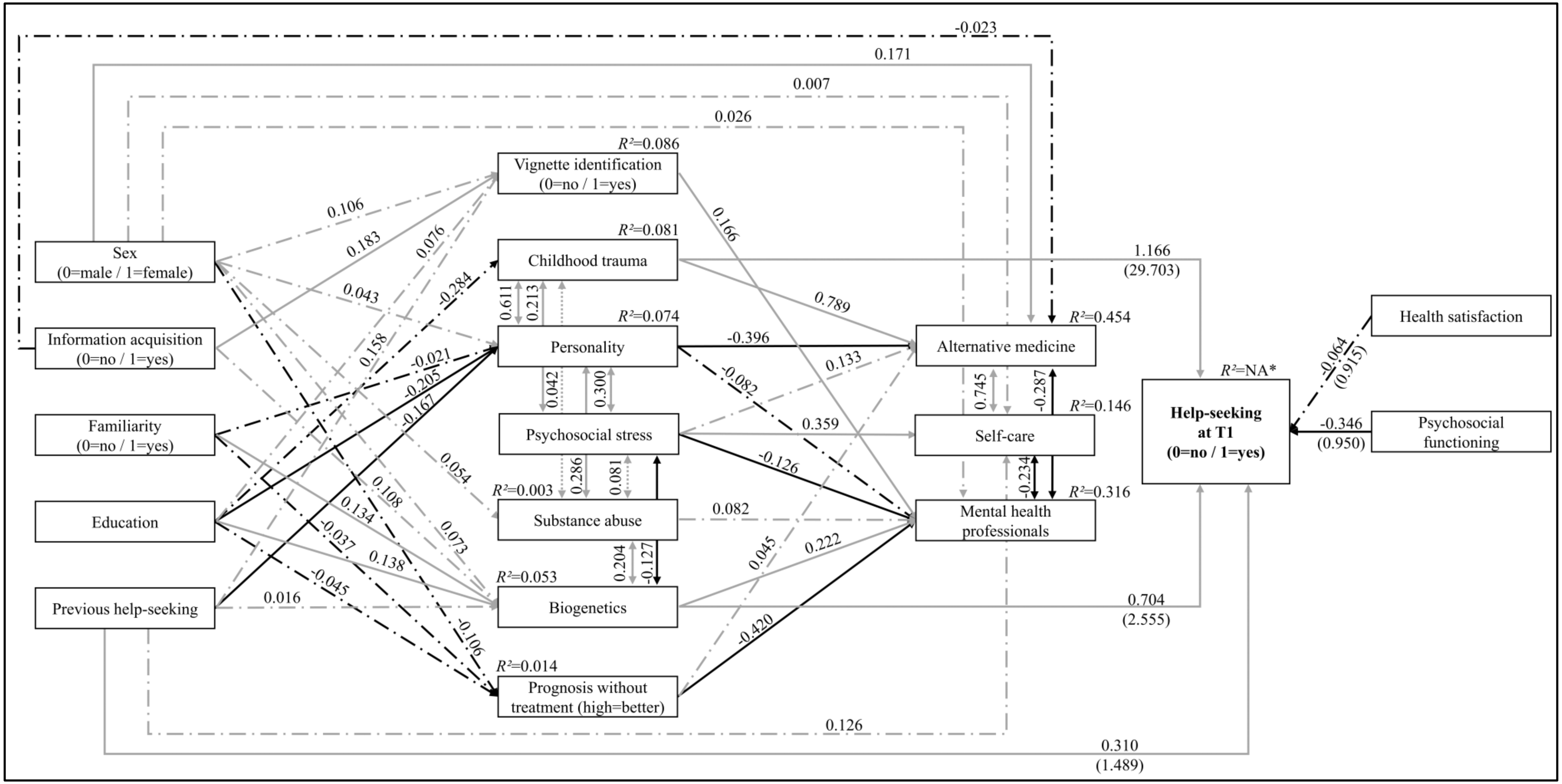

sFigure 11. Prospective model, only participants with mental health problems or disorders (*n*=217), with standardized path coefficients.

Model fit indices: $\chi 2$(82)=206.175 with $p<0.001$, CFI=0.924, SRMR=0.125, RMSEA=0.084 (90%CI=0.070-0.098). Power=0.889.

Odds ratios in brackets for the endogenous variable Help-seeking at T1. Solid lines indicate significant paths ($p\leq 0.05$), dashed and dotted lines indicate non-significant paths ($p>0.05$), grey indicates positive associations, black indicates negative associations. *R² not available because of empirical underidentification indicated by negative error variance.

References

1. McGlashan T, Walsh B, Woods S (2010) The Psychosis-Risk Syndrome: Handbook for Diagnosis and Follow-Up. Oxford University Press, New York

2. Schultze-Lutter F, Addington J, Ruhrmann S, Klosterkötter J (2007) Schizophrenia proneness instrument, adult version (SPI-A). Giovanni Fioriti Editore, Rome

3. Schultze-Lutter F, Marshall M, Koch E (2012) Schizophrenia proneness instrument child and youth version (SPI-CY): extended English translation (EET). Giovanni Fioriti Editore, Rome

4. American Association for Public Opinion Research (2016) Standard Definitions: Final Dispositions of Case Codes and Outcome Rates for Surveys. 9th edition. AAPOR, Alexandria

5. Schultze-Lutter F, Michel C, Ruhrmann S, Schimmelmann BG (2018) Prevalence and clinical relevance of interview-assessed psychosis-risk symptoms in the young adult community. Psychol Med 48:1167–1178. https://doi.org/10.1017/S0033291717002586

6. Michel C, Ruhrmann S, Schimmelmann BG et al (2018) Course of clinical high-risk states for psychosis beyond conversion. Eur Arch Psychiatry Clin Neurosci 268:39–48. https://doi.org/10.1007/s00406-016-0764-8

7. Schultze-Lutter F, Schimmelmann BG, Michel C (2021) Clinical high-risk of and conversion to psychosis in the community: A 3-year follow-up of a cohort study. Schizophr Res 228:616–618. https://doi.org/10.1016/j.schres.2020.11.032

8. Doll CM, Michel C, Betz LT et al (2021) The Important Role of Stereotypes in the relation between Mental Health Literacy and Stigmatization of Depression and Psychosis in the Community. Community Ment Health J. https://doi.org/10.1007/s10597-021-00842-5
